# Virulence Regulation and Innate Host Response in the Pathogenicity of *Vibrio cholerae*

**DOI:** 10.3389/fcimb.2020.572096

**Published:** 2020-09-30

**Authors:** Thandavarayan Ramamurthy, Ranjan K. Nandy, Asish K. Mukhopadhyay, Shanta Dutta, Ankur Mutreja, Keinosuke Okamoto, Shin-Ichi Miyoshi, G. Balakrish Nair, Amit Ghosh

**Affiliations:** ^1^Division of Bacteriology, National Institute of Cholera and Enteric Diseases, Kolkata, India; ^2^Global Health-Infectious Diseases, Department of Medicine, University of Cambridge, Cambridge, United Kingdom; ^3^Graduate School of Medicine, Dentistry and Pharmaceutical Sciences, Okayama University, Okayama, Japan; ^4^Collaborative Research Center of Okayama University for Infectious Diseases in India, National Institute of Cholera and Enteric Diseases, Kolkata, India; ^5^Microbiome Laboratory, Rajiv Gandhi Centre for Biotechnology, Thiruvananthapuram, India

**Keywords:** *V. cholerae*, virulence, toxins, quorum sensing, host response, microbiome

## Abstract

The human pathogen *Vibrio cholerae* is the causative agent of severe diarrheal disease known as cholera. Of the more than 200 “O” serogroups of this pathogen, O1 and O139 cause cholera outbreaks and epidemics. The rest of the serogroups, collectively known as non-O1/non-O139 cause sporadic moderate or mild diarrhea and also systemic infections. Pathogenic *V. cholerae* circulates between nutrient-rich human gut and nutrient-deprived aquatic environment. As an autochthonous bacterium in the environment and as a human pathogen, *V. cholerae* maintains its survival and proliferation in these two niches. Growth in the gastrointestinal tract involves expression of several genes that provide bacterial resistance against host factors. An intricate regulatory program involving extracellular signaling inputs is also controlling this function. On the other hand, the ability to store carbon as glycogen facilitates bacterial fitness in the aquatic environment. To initiate the infection, *V. cholerae* must colonize the small intestine after successfully passing through the acid barrier in the stomach and survive in the presence of bile and antimicrobial peptides in the intestinal lumen and mucus, respectively. In *V. cholerae*, virulence is a multilocus phenomenon with a large functionally associated network. More than 200 proteins have been identified that are functionally linked to the virulence-associated genes of the pathogen. Several of these genes have a role to play in virulence and/or in functions that have importance in the human host or the environment. A total of 524 genes are differentially expressed in classical and El Tor strains, the two biotypes of *V. cholerae* serogroup O1. Within the host, many immune and biological factors are able to induce genes that are responsible for survival, colonization, and virulence. The innate host immune response to *V. cholerae* infection includes activation of several immune protein complexes, receptor-mediated signaling pathways, and other bactericidal proteins. This article presents an overview of regulation of important virulence factors in *V. cholerae* and host response in the context of pathogenesis.

## Introduction

In many developing and underdeveloped countries, cholera remains a major public health problem. Historically, this disease is well-known for being associated with several large epidemics and pandemics. The causative agent of cholera, a Gram-negative bacterium *Vibrio cholerae*, has both environmental and human stages in its life cycle. This bacterium has a high capacity to adapt to varying conditions of salt concentration, pH, osmolarity and bile salts prevailing in the environment, and in human host. *V. cholerae* is classified into more than 200 somatic O antigen serogroups (Yamai et al., 1997). The O1 serogroup has two biotypes, classical and El Tor, both could individually be serotyped as either Ogawa or Inaba. The other toxigenic *V. cholerae* serogroup O139, emerged in the Indian subcontinent during 1992 and spread to other Asian countries (Ramamurthy et al., 2003). The rest of the serogroups are commonly known as *V. cholerae* non-O1, non-O139, or non-agglutinable vibrios (NAG). Apart from sporadic mild diarrhea, the non-O1/non-O139 serogroups of *V. cholerae* have also been found to be involved in invasive and extra-intestinal infections (Maraki et al., 2016; Zhang et al., 2020).

*V. cholerae* has several arsenal of virulence factors. Serotype switching, expression of toxins, biofilm formation, multiple transcriptional circuits, genome plasticity, adherence and invasions, cytolytic proteins, secretion systems, and the ability to respond to multiple stresses are some of the major determinants of *V. cholerae* pathogenecity. In addition to the interaction and association among all of these factors, the existence of multiple genetic and functional networks plays an important role in its pathogenesis. Bacterial pathogens have evolved mechanisms to sense the host environment and to adapt constantly to the specific niche they colonize, exquisitely regulating the production of specialized virulence factors (Ribet and Cossart, 2015). Expression of virulence factors to specific stimuli is controlled at the transcriptional and translational levels through intricate regulatory links. During chronic infection state, the bacterial regulatory genes are geared to sustain their fitness to adapt host conditions (Hindré et al., 2012; Damkiær et al., 2013). The innate host immune response to *V. cholerae* infection includes activation of the nuclear factor (NF)-κB, mitogen-activated protein kinase Toll-like receptor-mediated signaling pathways and other bactericidal proteins. This article provides a comprehensive review of the mechanisms involved in virulence of *V. cholerae* and the host immune responses it induces.

## Major Toxins Produced by *V. cholerae* and Their Regulation

### Cholera Toxin (CT)

**Figure 1:**
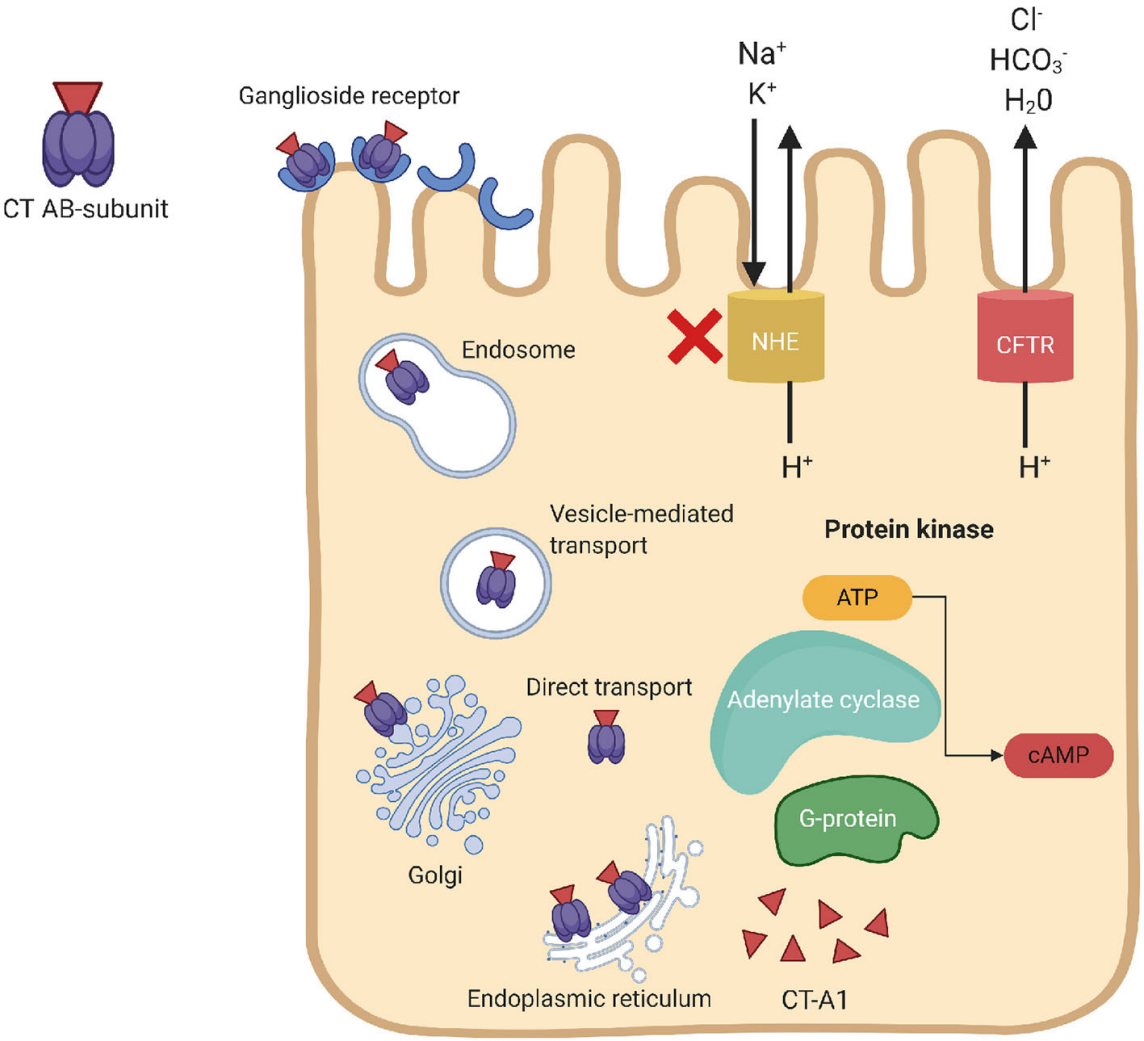
Mechanism of action of the cholera toxin. CT binds to the ganglioside receptor on the host epithelial cells, triggers endocytosis of the holotoxin. The internalized CT moves from the endosomes to the Golgi complex and endoplasmic reticulum (ER). The catalytic CT-A1 polypeptide transfers from the ER to the cytosol by retro-translocation through the action of the ER-linked degradation pathway to activate the Gsα subunit of guanine nucleotide-binding regulatory (Gα_*s*_) protein. Activation of Gα_*s*_-protein leads to increased adenylate cyclase (AC) activity, which cleaves ATP to cyclic adenosine monophosphate (cAMP) and subsequently activates protein kinase-A (PKA). Activation of PKA inhibits NaCl absorption through Na^+^/H^+^ exchanger (NHE transporters) and phosphorylate the cystic fibrosis transmembrane conductance regulator (CFTR) chloride channel proteins, which leads to ATP-mediated efflux of chloride ions and induce secretion of ${\text{HCO}}_{3}^{-}$, Na^+^, K^+^, and H_2_O. Loss of chloride ions induces massive fluid secretion in the small intestine, deposing the resorptive ability of the large intestine, which results in severe watery diarrhea.

Cholera toxin is the main virulence factor of *V. cholerae*, which is composed of one A subunit (toxic domain) and five B subunits (receptor-binding domain). The basic mechanism of action of CT is shown in Figure 1. Secreted CT-B binds to monosialoganglioside (GM1) on the surface of host cells to facilitate internalization of CT-A that prompts fluid loss *via* cAMP-mediated activation of anion secretion and inhibition of electroneutral NaCl absorption. The action of the barrier-disrupting effects of CtxA with massive Cl^−^ secretion leads to the severe diarrhea, which is characteristic of cholera.

While there is no variation in the CT-A subunit, CT-B has several amino acid substitutions and some are specific to biotypes (Ramamurthy et al., 2019). These AA residues do not take part in binding to GM1 and hence unlikely to influence affinity for the receptor. On the other hand the AA changes might influence toxin immunity and hence play an important role in the severity of the infection. The gene encoding the CT (*ctx*) is located in the CT cassette, which has been shown to be a prophage (CTXΦ) that could integrates in the *V. cholerae* chromosome using Tcp (toxin-coregulated pili) as a receptor (Davis and Waldor, 2003). However, *V. cholerae* O139 uses mannose-sensitive hemagglutinin (MSHA) pilus as a receptor VGJFΦ or its satellite phage RS1 (Campos et al., 2003). Hence, strains that are not expressing the Tcp may use other mechanisms to acquire CTXΦ. The typical genome of CTXΦ consists of the core and RS2 regions. The core region is constituted with seven genes, *psh, cep, gIIICTX, ace, zot, ctxA*, and *ctxB*. Except for *ctxA* and *ctxB*, rest of the genes in the core region are involved in phage morphogenesis. RS2 region contains three genes, *rstA, rstB*, and *rstR*, which are associated with CTXΦ replication, integration and regulation, respectively (Waldor et al., 1997). In addition, an antirepressor RstC located within the RS1 promotes the expression and transmission of CTXΦ genes. The regulatory aspects of CT have been discussed in the ToxRST system. Apart from ToxR, a three-component signal transduction system VieSAB was shown to enhance the CT expression indirectly through controlled ToxT expression (Tischler et al., 2002). However, the role of environmental factors controlling this signal system has not been established.

### Accessory Cholera Enterotoxin (Ace) and Zonula Occludens Toxin (Zot)

Apart from CT, accessory cholera enterotoxin (Ace) and zonula occludens toxin (Zot) are present in the core region and contribute to *V. cholerae* pathogenesis by inducing changes in the intestinal barrier. The genes encoding them are present in the N-terminal side of the core region, which is involved in CTXΦ morphogenesis (Pérez-Reytor et al., 2018). Ace is an integral membrane protein that stimulates Ca^2+^ -dependent Cl^−^/HCO^3−^ cotransporters, induces fluid secretion in the rabbit ileal loop and alters short-circuit current (*I*_sc_) in the Ussing chambers (Somarny et al., 2002). The process of secretion by Ace involves Ca^2+^ as a second messenger, as there is no secretory response to cAMP or cGMP agonists (Trucksis et al., 2000). It appears that Ace may cause initial intestinal secretion *in vivo* during *V. cholerae* infection before the slow action of CT. Anoctamins (ANOs) are the transmembrane protein on the cell surface, which are essential for the calcium-dependent exposure of phosphatidylserine. The role of ANOs in diarrhea is well-investigated in NSP-4 of rotavirus. It was found that phosphatidylinositol 4,5-bisphosphate (PIP_2_) influences the ANO6 function by Ace stimulation in intestinal epithelium for Cl^−^ secretion to induce diarrhea (Aoun et al., 2016). ANO6 and PIP_2_ act as additional mechanisms of secretory diarrhea. Overall, a comprehensive study on the role of Ace from the pathophysiological point of view is still lacking.

Zot is involved in the CTXΦ morphogenesis and its promoter activity has been identified within the *ace* sequence. Zot affects the structure of epithelial tight junction (TJs) of the small intestine. This modification leads to increase of mucosal permeability, resulting passage of macromolecules through the paracellular route. Binding of Zot to the human α-1-chimaerin receptor, which is a neuron-specific GTPase-activating protein that induces a reduction in epithelial electrical resistance and increases the trans-epithelial flux and permeability of TJs (Uzzau et al., 2001). This binding modifies the cytoskeleton and the TJ complex inside of the cell *via* an intracellular signaling that reduces the actin filaments by changing the F- and G-actin pools. This action increases intestinal epithelial permeability by affecting the TJs (Goldblum et al., 2011). Though the TJ molecular structure and its assembly during morphogenesis are well-studied, its physiological regulation and response to toxins still remains incomplete.

### Heat-Stable Enterotoxin (ST)

**Figure 2:**
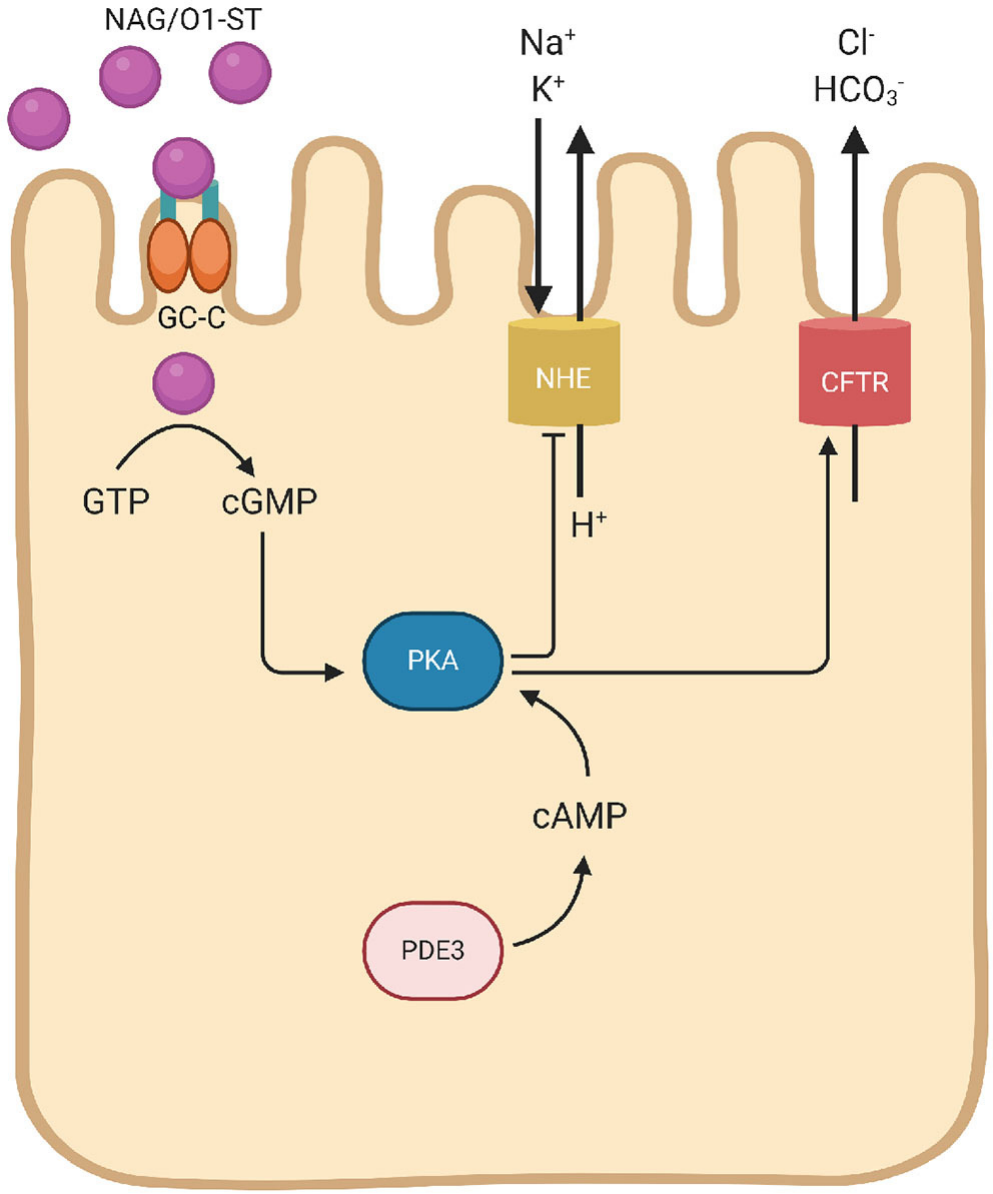
Schematic mechanisms of *V. cholerae* heat-stable eterotoxin (NAG/O1-ST). NAG/O1-ST bind to the intestinal guanylate cyclase (GC-C). Activation of intracellular catalytic domain of GC-C result in the formation of cyclic guanosine monophosphate (cGMP) from guanosine triphosphate (GTP). This intracellular transformation activates cGMP-cAMP-dependent PKA leads to CFTR phosphorylation. cGMP reduces Na^+^ and Cl^−^ absorption through the NHE, and also inhibits phosphodiesterase-3 (PDE3) leading to cellular accumulation of cAMP, and subsequent activation of PKA. Phosphorylation of the CFTR leads to secretion of Cl^−^ with ${\text{HCO}}_{3}^{-}$ and decreased NaCl absorption, which results in diarrhea.

Some strains of *V. cholerae* elaborate heat-stable enterotoxin (ST) that signal cyclic guanosine monophosphate (cGMP) pathways to activate the cystic fibrosis transmembrane conductance regulator (CFTR) chloride channel, and elevates intracellular cGMP to induce anion secretion and diarrhea (Figure 2). ST expression by *V. cholerae* O1 strains is rare, but is mostly reported in enterotoxigenic *Escherichia coli* and NAGs. The basis of NAG-ST expression by environmental strains of *V. cholerae* is not known. NAG/O1-ST binds to guanylyl cyclase C (GC-C) on the apical surface of enterocytes, signals intracellular cGMP, and a cGMP-dependent kinase (PGKII) to phosphorylate CFTR on the apical membrane. The activation of cGMP and CFTR signaling stimulates chloride and fluid secretion (Al-Majali et al., 2000). PGKII plays an important role in regulating cGMP-dependent translocation of CFTR and ST-dependent anion secretion, which is independent of CT regulation (Golin-Bisello et al., 2005). It has been seen in *E. coli* STa, that GC-C is also implicated in the regulation of the intestinal pH, as CFTR transport Cl^−^ as well as ${\text{HCO}}_{3}^{-}$ to prevent the tissue damage. This ${\text{HCO}}_{3}^{-}$ secretion is unaided either by PKGII or CFTR (Weiglmeier et al., 2010). *E. coli* STp is encoded within a transposon (Tn1681) flanked by inverted repeats of insertion sequence 1 (IS*1*) and hence able to spread widely by gene transfer. The flanking regions of gene encoding the NAG-ST lack any transposon, but has direct repeats (DRs). DRs are known for chromosomal rearrangements such as transpositions, duplications, and deletions.

### Cholix Toxin (Chx)

Cholix toxin is a member of the diphthamide-specific class of ADP-ribose transferases that has a specific ADP-ribose transferase activity against ribosomal eukaryotic elongation factor-2 (eEF2) (Jørgensen et al., 2008). Chx has been mostly identified in environmental *V. cholerae* non-O1, non-O139 strains. Though lipoprotein receptor-related protein has been shown to bind with Chx, the specific receptor for cholix bining is not known, as the sensitivity of human cell lines to cholix is variable. Recently, prohibitin (PHB) was identified acts as a Chx-binding protein (Yahiro et al., 2019), which is expressed by some cell membranes, and also by mitochondria and nucleus. *V. cholerae* ChxA has some homology with exotoxin A (ToxA) of *Pseudomonas aeruginosa*, but there is no evidence for lateral transfer (Purdy et al., 2010). In addition, *chxA* gene present on chromosome 1 (between VC1644 and VC1645) is not flanked by phage-like sequences or IS elements.

**Figure 3:**
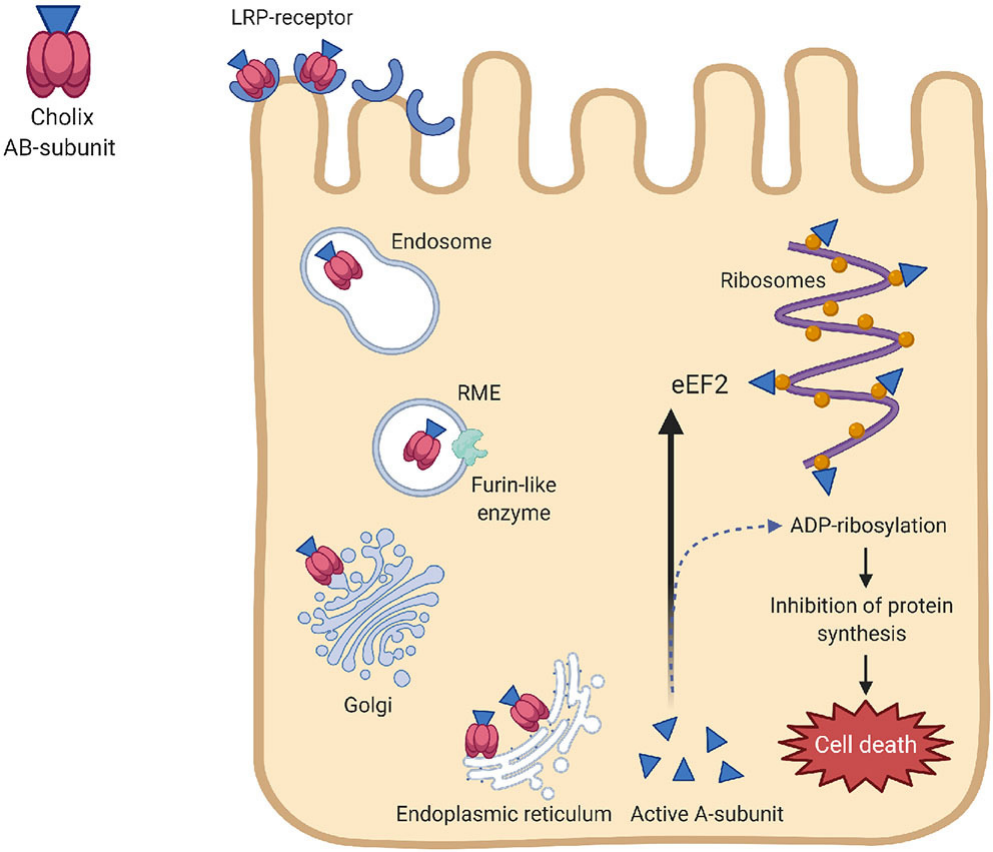
Mechanisms of action of cholix toxin. Chx binds to the lipoprotein receptor-related protein (LRP-receptor) of the eukaryotic cells, followed by internalization by receptor-mediate endocytosis (RME). A furin-like enzyme is believed to be responsible for nicking cholix in its arginine-rich loop. After passing the Golgi complex, cholix follows the retrograde pathway in the endoplasmic reticulum to form the A and B-subunits. The A-subunit translocate into the cytoplasm and prevents protein synthesis by altering the diphthamide residue of elongation factor 2 (eEF2) through its ADP-ribosylation activity. Inhibition of protein synthesis by specific modification of eEF2 leads to cell death.

The mechanism of action of Chx is shown in Figure 3. Chx transports across intestinal epithelium through a vesicular trafficking pathway that rapidly reaches vesicular apical to basal transcytosis by avoiding the lysosomes (Ogura et al., 2017). This toxin has the necessary attributes required for the infection of host cells by receptor-mediated endocytosis, translocation to the cytoplasm, and inhibition of protein synthesis by specific modification of eEF-2. Transfer of an ADP-ribose group from NAD ^+^ to a diphthamide in eEF2 inhibits protein synthesis leading to cell death (Jørgensen et al., 2008). The pathways responsible for Chx-induced hepatocyte (HepG2) death involves reactive oxygen species (ROS) and MAPK-dependent effects (Ogura et al., 2017). Chx interacts with PHB and induces mitochondrial dysfunction and cytoskeletal rearrangement by rho-associated coiled-coil-containing protein kinase-1 activation during apoptosis. This alters the respiratory supercomplexes formation, followed by elevation of ROS-production. However, the role for cholix in causing diseases has not been clearly proven. Of the three types of Chx (ChxA-I, ChxA-II, and ChxA-III) detected in *V. cholerae*, chxA-I and ChxA-II have been shown to cause severe damage to internal organs in mice, which suggests the involvement of cholix in extraintestinal infections (Awasthi et al., 2013).

### Multifunctional Autoprocessing Repeats-in-toxin (MARTX Also Known as RTX)

RTX is one of the pore-forming accessory toxins that translocates specific effector molecules into a target eukaryotic cell to carry out distinct functions. The RTX toxins comprise proteins encoded by four genes (*rtxACBD*) in two operons, located adjacent to the *ctxAB* on the large chromosome: *rtxA* encoding the toxin; *rtxB/rtxE*, an ATP-binding cassette transporter of RtxA; *rtxC*, an acylase of RtxA; and *rtxD*, with unknown function (Linhartová et al., 2010). These distinct virulence activity proteins are responsible for the RTX effect. RTX is produced both by clinical and environmental strains of *V. cholerae*. Early studies showed that the non-toxigenic *V. cholerae* O1, exerts cell rounding effect by using the RTX that causes depolymerization of actin stress fibers and covalent cross-linking of cellular actin into multimers targeting the G-actin (Fullner and Mekalanos, 2000). This direct catalyzation of RTX leads to covalent cross-linking of monomeric G-actin into oligomeric chains that cause permanent disassembly of the cytoskeleton (Cordero et al., 2006). *rtxA* gene is one of the largest ORFs of the *V. cholerae* genome. The *rtx* locus is linked to the CTXΦ integration site and the putative repressor that regulates *rtxBDE* lies outside of the *rtx* locus. In classical biotype strains, there is a deletion at the 5′ end of the *rtxA* gene along with the *rtx* promoter region. This deletion affects RTX toxin maturation and secretion deactivates the RTX.

RTX is exported to the extracellular milieu by an atypical type 1 secretion system (T1SS) that disrupts the actin cytoskeleton followed by inactivation of small Rho GTPases, Rho, Rac, and Cdc42 (Kudryashov et al., 2008; Prochazkova and Satchell, 2008). This T1SS regulation of RTX at the transcriptional level is independent of quorum sensing (QS). In certain strains of *V. cholerae*, QS indirectly regulates the RTX activity. RTX is autoprocessed by an internal cysteine protease domain (CPD), which is activated by the eukaryote-specific small molecule inositol hexakisphosphate (Lupardus et al., 2008). Under *in vitro* conditions, the exported bacterial proteases can damage the RTX activity and thus play a role in its own growth phase regulation. Presence of CPD proteolytically activates the RTX in *V. cholerae* for colonization in intestinal epithelial cells. In addition, the host response to bacterial factors also reduces the RTX toxicity, resulting in a non-inflammatory diarrheal disease response (Woida and Satchell, 2020).

### Hemagglutinin Protease (HAP)

Hemagglutinin protease expression is a stationary phase growth-specific system and induces a hemorrhagic response with symptoms like necrosis, acute myofibre degeneration and degeneration of laminin, and collagen of vascular endothelial cells. HAP is secreted through the type II secretion (T2SS) pathway of *V. cholerae*. Expression of *hapA* is complex that involves a combination of several environmental signals through many global regulators, including cyclic AMP (cAMP) receptor protein (CRP), and RNA polymerase sigma (RpoS) subunit (Silva and Benitez, 2004). The RNA polymerase-binding transcription factor (DksA)-HapR-RpoS axis regulates hemagglutinin protease production in *V. cholerae* (Basu et al., 2017). HapR is expressed at high cell density. Repression of *hapR* takes place at low cell density, a condition conducive for expression of *aphA*. By binding to their respective promoter regions, HapR directly repress *tcpP, tcpA*, and *toxT* transcription in an indirect mode *via* AphA and a ferric uptake regulator (Fur) and directly activates the transcription of *tcpP, toxT*, and *tcpA*. However, the mechanisms of the Fur-dependent activation of Tcp expression needs further investigation. HapR and Fur seemed to have no regulatory actions on *toxR* transcription.

Endogenous nitric oxide (NO) production modulates HAP-mediated cytotoxicity. HAP causes degradation of the mucus barrier, modification of some of the toxins and also acts on TJ-associated proteins (Wu et al., 2000). Cleavage of occludin by HAP causes a reorganization of one of the zonulae occludents, the F-actin cytoskeleton and disruption of paracellular barrier function (Benitez and Silva, 2016). CRP is important for the expression of multiple HapR-regulated genes (Liang et al., 2007). Inactivation of *crp* represses *ompU, ompT*, and *ompW* encoding outer-membrane proteins, the alternative sigma factor (σE) required for intestinal colonization and genes involved in anaerobic energy metabolism. Most strains of *V. cholerae* O1 and NAGs carry a gene *hlyA*, which codes for hemolysin (HlyA). Production of HlyA is controlled by QS that is regulated by transcription factor HapR, at two levels-at the transcription level, independent of the metalloprotease HapA and also at the post-translational level mediated by HapA (Tsou and Zhu, 2010).

HAP can potentially contribute to the nicking and activation CT-A subunit and process pro-HlyA to active and mature form of HlyA during infection at low cell density. In addition, HAP supports long persistence of *V. cholerae* in the small intestine, delivery of CT in the vicinity of its receptor, proteolysis of biofilm protein RbmA that increase cell-to-cell adhesion by supporting interaction between biofilm cells and planktonic cells, controlling the VarS/VarA-CsrA/B/C/D sensory system. Thus, HAP has multiple targets during infection to increase the severity of the infection.

### Mannose-Sensitive Hemagglutinin (MSHA)

The mannose-sensitive hemagglutinin (MSHA) is a member of the family of type 4 pili, which is important as host colonization factors, bacteriophage receptors, and mediators of DNA transfer in Gram-negative bacteria. Presence of MSHA pilus is another phenotypic marker for the El Tor vibrios and is associated with biofilm formation and environmental survival of *V. cholerae*. MSHA locus is organized as two operons and many of the genes located upstream of the *mshA* and involved in the secretion and assembly of MSHA pilin subunits. Presence of a 7-bp direct repeat flanking *msh* suggests that the MSHA gene locus may have been acquired as a transposable or a mobilizable genetic element.

*V. cholerae* with MSHA phenotype become inactive in the intestine due to the binding of host's secretory immunoglobulins. In order to establish successful gut colonization, Tcp represses *msh* genes while activating *tcp* genes during infection (Hsiao et al., 2008). The ability of *V. cholerae* to regulate this switch over process determines its survival in the host. The two pili systems are intertwined post-transcriptionally through the ToxT-regulated pre-pilin peptidase that degrades MshA in a TcpJ-dependent manner. Expression of the MSHA biosynthesis is directly regulated by ToxT by binding to three different promoters within the *msh* locus. *V. cholerae* binds S-IgA in an MSHA-dependent and mannose-sensitive manner that prevents bacteria from crossing mucus barriers and attaching to the surface of epithelial cells (Hsiao et al., 2008).

### *V. cholerae* Cytolysin (VCC)

*V. cholerae* cytolysin (VCC) is a member of the β-barrel pore-forming toxin (β-PFT) family and has mostly been reported in non-toxigenic strains. VCC has membrane-damaging cell-killing activity and acts on the target cells by making transmembrane oligomeric β-barrel pores leading to permeabilization of the target cell membranes (Rai and Chattopadhyay, 2015). Encoded in the *hlyA* gene of *V. cholerae*, VCC initiates mitochondria-dependent apoptosis due to its anion channel activity through several signal transduction pathways (Kanoktippornchai et al., 2014). These anion channels in the apical membrane of enterocytes trigger an outer transcellular flux of Cl^−^. This ion movement, associated with the external movement of Na^+^ and water is responsible for the diarrhea caused by the non-toxigenic strains of *V. cholerae*. The QS regulator HapR, along with Fur and HlyU has been shown to regulate the transcription of *hlyA* in El Tor vibrios (Gao et al., 2018). This complex regulation helps *V. cholerae* in invasion and pathogenesis during the different infective stages. VCC pro-inflammatory responses significantly rest on the activation of the transcription factor nuclear factor-κB and mitogen-activated protein kinase (MAPK) family (Khilwani et al., 2015).

This pore formation by VCC needs the presence of cholesterol in the liposome membranes. Specific binding of VCC with the membrane lipid components using the distinct loop sequences within the membrane-proximal region of VCC is considered to play a key role in determining the efficacy of the pore-forming process. Membrane binding of VCC, oligomerization, and pore-formation activity are facilitated by several regulatory mechanisms and physicochemical factors, which are not fully established. It was observed that in comparison with freely secreted VCC, VCC-associated with outer membrane vesicles (OMVs), which might play a role in its stability enhanced its biological activities (Bitar et al., 2019). OMV-associated VCC triggers an autophagy response in the target cell, which acts as a cellular defense mechanism against an OMV-associated bacterial virulence factor. VCC causes extensive vacuolation and death of cultured cells and forms an anion-selective channel in planar lipid bilayers and in cells. The formation of the anion channel is important for the progression of the vacuoles and for the cell death induced by VCC (Moschioni et al., 2002).

## Control of Virulence by the ToxR Regulon

**Figure 4:**
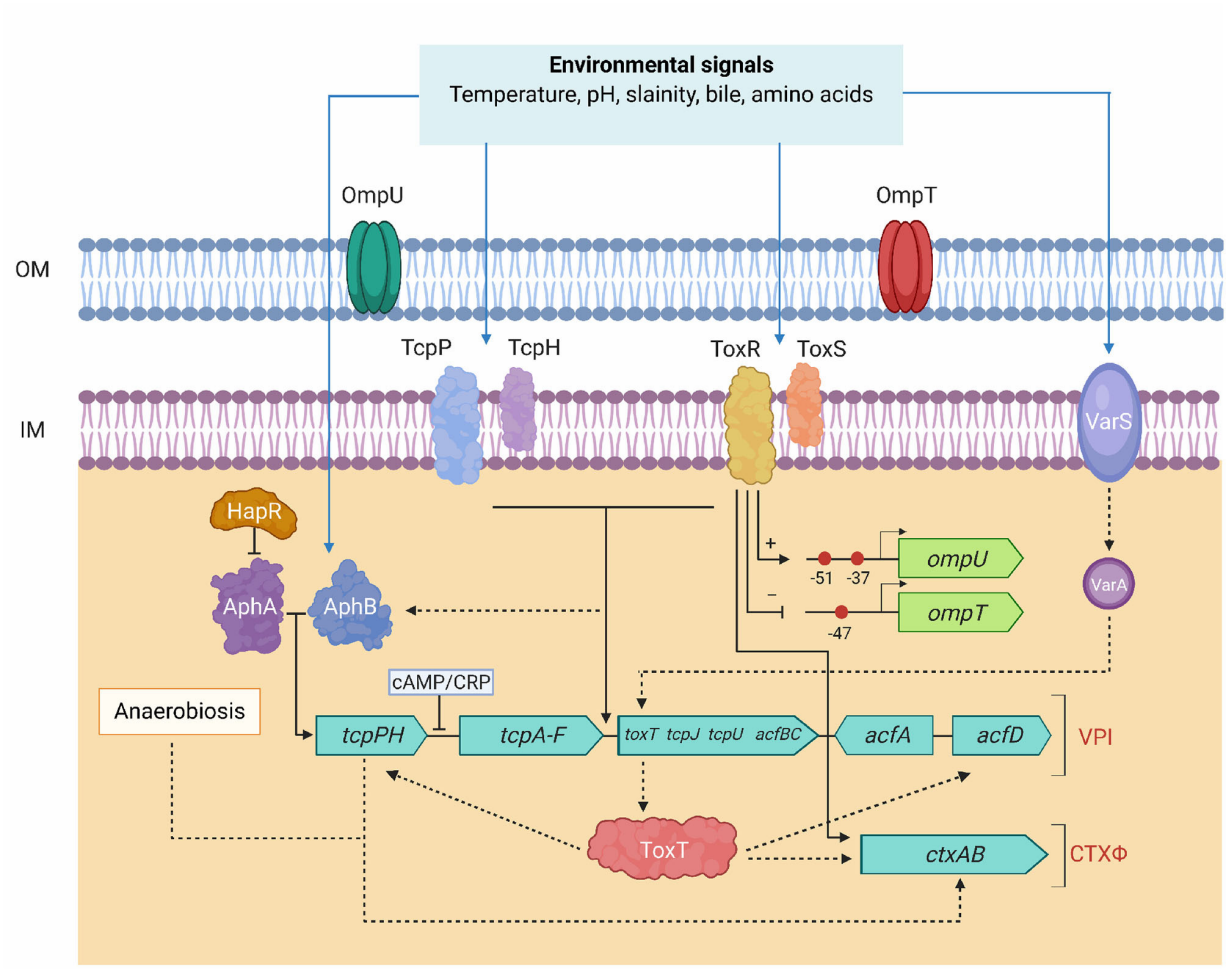
Regulation model of Tox-mediated virulence genes in *V. chlerae*. TcpP/TcpH and ToxR/ToxS function together in activating transcription of *toxT*. ToxR and TcpP need accessory proteins (ToxS and TcpH, respectively) for maximal activity. ToxT initiates transcription of the full *tcp* operon, *ctxAB*, and *acf*. AphA and AphB activate transcription of the *tcpPH* operon in response to environmental conditions. *tcpPH* promoter is negatively influenced by the global regulator, CRP. ToxR/ToxS, and AphA/AphB also regulate genes other than *ctx* and *acf*. ToxRS, AphAB, TcpPH, and ToxT coordinately regulate the transcription of *ctx* and *tcp*. AphA and AphB activates expression of the *tcpPH*. TcpPH and ToxRS regulate *ctx* and *tcp* genes through ToxT. AphB has been linked to the expression of *tcpPH* for anaerobiosis that enhances the production of CT. The direct activator and the second activator are differentiated by black solid and dotted arrows, respectively. Blue arrows indicate different environmental conditions sensed by the ToxRS and TcpPH, AphAB, and VarS regulatory systems. The two-component VarS-VarA system responds to environmental factors and signals *toxT*. ToxR, independently activates and represses transcription of *ompU* and *ompT*. ToxR modulation was shown by an arrow (*ompU*) and line with base (*ompT*) through + for the transcription of *ompU* and—for repressing the transcription of *ompT*. Red circles in *ompU* and *ompT* indicate the ToxR binding sites and the transcriptional start sites are marked with arrows for each promoter.

ToxR is a DNA-binding protein and a member of the outer membrane protein regulator-R (OmpR) subclass of two-component activator (TA) systems. ToxR tightly regulates expression several virulence encoding genes in response to environmental stimuli and also plays an important role in de-repressing genes that are silenced by H-NS. It coordinately regulates the expression of a number of genes aided by the products of two other genes, *toxS* and *toxT*. Figure 4 shows involvement of multiple signaling facilitated by ToxR, ToxS, and ToxT in controlling expression virulence genes. OmpT, a dominant outer membrane porin regulated by *V. cholerae* in response to different environmental factors. ToxR directly repress the *ompT* promoter and activate the *ompU* promoter, but involves a second activator, TcpP, to initiate the *toxT* promoter that encodes the transcription factor. Modulation of OmpU and OmpT is critical for *V. cholerae* bile resistance, virulence factor expression, and intestinal colonization (Provenzano and Klose, 2000; Morgan et al., 2019). Strains expressing only the OmpT show considerably reduced *in vitro* expression of virulence factors and intestinal colonization (Provenzano and Klose, 2000). It was also found that two transcriptional regulators, cAMP receptor protein (CRP) and ToxR, compete in the *ompT* promoter region. ToxR functions as an antiactivator and repressor, depending on its interplay with CRP, which activates *ompT* transcription by a loop-forming mechanism (Song et al., 2010). In classical vibrios, ToxR directly induce CT expression independent of ToxT and the presence of bile salts enhances this activation. The antimicrobial bactericidal/permeability-increasing (BPI) protein and a BPI-derived peptide P2 kills invading pathogens by increasing outer membrane permeability and inhibiting the O_2_ consumption. ToxR regulates the outer membrane protein OmpU so as to confer resistance to P2. It was demonstrated that *V. cholerae* lacking *toxR* is sensitive to P2 than is wild type strain (Mathur and Waldor, 2004).

ToxR and ToxS function as direct mediators of signal transduction by detecting signals with their periplasmic domains and control transcription of regulated genes with their cytoplasmic domains. For full activity, ToxR requires ToxS, a periplasmic integral membrane protein, which stabilizes the ToxR by protecting it from premature proteolysis. ToxRS interact through their corresponding periplasmic domains, ToxRp and ToxSp (Midgett et al., 2017). For gut colonization and pathogenesis, *V. cholerae* has to recognize and respond to the environmental signals and cell density to ensure proper expression of genes. The molecular mechanism of cell-density dependent expression of *toxR*, needs further investigation. Bile sensory system facilitates the ToxRS complex to achieve this (Mey et al., 2015; Lembke et al., 2018). ToxR activation by bile salts depends on the function of ToxS. Bile inhibits ToxR degradation under starvation and alkaline pH condition or when the serine protease DegPS acts on the reduced disulfide bonds. In the subsequent stage, bile is coupled with ToxRS complex and it triggers transcription (Thomson and Withey, 2014). Even though the basic functional hierarchy of the ToxR regulon is known, there are still certain gaps that need to be addressed clearly. For instance, CT and Tcp are both induced by the same pathway, but can be expressed differently. Similarly, it is not clear how a different expression of ToxR regulon components affects the virulence factors during colonization of *V. cholerae* in the host.

The second transcriptional activator, ToxT, is positively regulated by ToxR. Coordinated expression of virulence genes is regulated by the ToxR, TcpP, and ToxT proteins (Figure 4). The amino-terminal region of TcpP has a sequence homology to the DNA-binding domains of many regulatory proteins, including ToxR. Pairs of ToxT-binding sites in the promoters are important for dimerization of ToxT, which facilitate the binding of DNA and activate the expression of virulence genes. Depending on the structure of the promoter, ToxT can function as a monomer or a dimer. ToxR and TcpP are the two inner membrane proteins that activate transcription of *toxT* and ToxT directly initiates virulence gene expression, especially the CT and Tcp. In response to human host signals, TcpP also stimulates virulence factors. ToxT is also responsible for the activation of putative accessory virulence genes, such as *aldA* (aldehyde dehydrogenase), *tagA* (ToxR-activated gene-A), *acfA,D* (accessory colonization factor), and *tarAB* (ToxT-activated RNA) (Thomson and Withey, 2014). ToxT activity is negatively modulated by bile and unsaturated fatty acids existing in the upper small intestine and the presence of bicarbonate in the same milieu increases the ToxT binding affinity raising the level of virulence gene transcription. Anaerobiosis has been shown to affect the production of virulence factors. Aerobic respiration control protein (ArcA) detects the signal of low oxygen to enhance biofilm formation. ArcA may upregulate virulence gene expression under the anaerobic condition by activating *toxT* expression (Sengupta et al., 2003). However, influences of environmental factors responsible for the activation of virulence system through ToxT have not been elucidated.

ToxR is not a direct activator in the *toxT* expression system but, it enhances the activity of TcpP, perhaps by recruiting it to the *toxT* promoter (Krukonis et al., 2000). TcpP/TcpH comprise a pair of regulatory proteins that have functional similarity with ToxR/ToxS and these regulatory proteins are required for *toxT* transcription. ToxR directly activates the porin encoded *ompU* promoter along with TcpP as a second activator to initiate the transcription factor, which activates virulence factors including CT and Tcp (Morgan et al., 2011). In association with TcpH, TcpP form TcpP/TcpH transcription activation complex and activate downstream virulence gene expression grouping with ToxR/ToxS complex. Of the four cysteine residues present in the cytoplasmic domain of TcpP, C58 is essential for its function and also for activation of virulence gene expression and gut colonization in infant mice model (Shi et al., 2020). Currently, there is no evidence that Tcp directly binds to intestinal cells. In addition, the interaction between Tcp and a specific receptor on host cells has not been established.

Expression of genes encoding the Tcp differs between classical and El Tor biotypes. Intergenic regions between *tcpI* (methyl-accepting chemotaxis protein) and *tcpP* (trans-cytoplasmic membrane protein), and between *tcpH* (periplasmic or exported protein) and *tcpA* (cytoplasmic membrane protein) have substantial sequence differences in these two biotypes. These sequence differences in *tcp* influence their response to pH and temperature signals. AphB, which is the activator of *tcpP* and *tcpH*, belongs to the family of LysR transcriptional regulators and along with AphA plays a role in the differential regulation of virulence genes in classical and El Tor vibrios by activating ToxR virulence cascade that leads to the transcription of the *tcpPH* operon in response to any environmental stimuli. The global regulator, CRP represses tcpPH transcription *via* its ability to influence AphA and AphB-dependent transcriptional activation. This is likely because the CRP binding site is completely within the binding sites of AphA and AphB. A single base-pair change at positions−65 and−66 of *tcpPH* promoters was shown to be responsible for the differential regulation of virulence gene expression in classical and El Tor vibrios, respectively (Kovacikova and Skorupski, 2000). Reciprocal interchange of the *tcpPH* promoter between the two biotypes showed that their ability to activate the transcription is essentially dependent on the *tcpPH* promoter with the presence of either an A or a G at position−65 or−66 in classical and El Tor, respectively. Interaction of ToxRS, ToxT, TcpPH, and other virulence related proteins/genes in are shown in Table 1. Several *in vitro* studies have shown the role of complex ToxR regulatory network in modulating the expression of virulence genes, but their coordinated regulatory responses during infection process still remain largely unknown.

**Table 1:** Interaction of ToxRS, ToxT, TcpPH, and other virulence related proteins/genes in *V. cholerae*.

**Protein**	**Action/significance**
ToxR/S	Interaction of ToxR with ToxS is required for full transcriptional activation by enhancing dimerization of ToxR. ToxR is protected from degradation and alkaline pH by ToxS. break ToxRS detect and transduce signals into transcriptional regulation programs.
	Activation of CT, Tcp, Acf through ToxT. ToxR act as a coactivator by increasing transcriptional activation of *toxT* by promoting TcpP recruitment and/or binding to the *toxT* promoter.
	Directly repress *ompT* expression and activate *ompU* transcription that changes the outer-membrane porin composition. break Upregulation of OmpU is important for resistance to bile acids and antimicrobial peptides present in the host.
	Enhances TcpP binding and activation of transcription.
	ToxR-regulated *tcpI* and *acfB* function with a two-component system to regulate chemotaxis.
ToxT	Activate about eight different virulence gene promoters, including the *ctxAB, tcpA* and *acf* promoters, as part of a virulence gene regulatory cascade.
	Transcription is influenced by bile, unsaturated fatty acids and bicarbonate. break Influenced by environmental signals through VarS and VarA.
	Represses expression of MSHA.
	Transcription is regulated by ToxRS and TcpPH.
	H-NS directly represses the expression of the *toxT*.
TcpP	Transcription is stimulated by AphAB.
	Temperature and pH influence the levels of TcpP through intramembrane proteolysis.
	Activation is signaled by HapR.
TcpH	Required for stability of TcpP and co-expressed with *tcpP* as an operon. break TcpH protect TcpP against this proteolytic degradation.
AphA/B	AphA activate transcription in the presence of AphB.
	Synergistic function of AphAB activates *tcpPH* transcription.
	Alteration of dyad symmetry due to base pair changes in the *tcpPH* promoter and binding of AphB is responsible for the differential expression of virulence genes in *V. cholerae* classical and El Tor biotypes.
	The promoters directly activated by AphB respond to intracellular pH and anaerobiosis during the early stages of the infection.
	A main transcriptional activator of biofilm formation, virulence genes, and pH homeostasis.
	HapR represses transcription.

## Regulation of Secretion Systems (T2SS/T3SS/T6SS)

Secretion systems are used to transport macromolecules across the membranes. Some of the factors such as motility (required for successful colonization), intestinal colonization and expression virulence are dependent on the secretion of effector molecules in the cellular environment and host cytoplasm. Of the eight types of secretion systems that described, T2SS, T3SS, and T6SS are well-studied in *V. cholerae*.

The T2SS, also known as extracellular protein secretion (Eps) system, is involved in transport of hydrolytic enzymes and may play a role in ameliorating the cellular environments and generating nutrients to support bacterial fitness. T2SS secreted proteins and protein complexes are folded and accumulated in the periplasm of the bacterial cells. The T2SS selectively translocates toxins and several enzymes in their folded state across the outer membrane. CT is translocated as a folded protein complex from the periplasm across the outer membrane through the T2S channel and is captured within the large periplasmic vestibule of *V. cholerae* general secretory protein-D (VcGspD) before its secretion (Reichow et al., 2011). The other T2SS secreted proteins, such as *V. cholerae* extracellular *s*erine proteases (VesA), Hap, and sialidase are functionally associated with CT (Sikora et al., 2011). The pilin-like proteins of T2SS assemble into a pseudopilus and are exported by the extracellular polysaccharide synthesis protein (EpsD), which is an itegral outer membrane pore and also helps in exclusion of intact CTXphi from *V. cholerae* (Yanez et al., 2008; Faruque and Mekalanos, 2012). Proteases present in the OMVs of *V. cholerae* also play a role in its pathogenesis. It is believed that QS plays an important role in directing the activation of the T2SS and the initiation of exoprotein release under favorable environmental conditions (Sandkvist, 2001). Using the T2SS, *V. cholerae* produces calcium-dependent trypsin-like serine protease (VesC) and Zn-dependent HAP, which is important in the initial phase of intestinal colonization, hemorrhagic fluid response, necrosis, increased interleukin-8 (IL-8) response, and apoptosis (Mondal et al., 2016).

The second messenger nucleotide cyclic dimeric guanosine monophosphate (c-di-GMP) regulates T2S, however, the range of phenotypes regulated by c-di-GMP is not fully known. At least for biofilm life style of *V. cholerae*, it is established that high intracellular c-di-GMP concentration has a strong association. T2SS contains 13 proteins, of which 12 are encoded by the extracellular protein secretion (*eps*) gene cluster. VpsR seems to have a molecular-link involving the intracellular c-di-GMP concentration to both *Vibrio* polysaccharide (Vps) biosynthesis and T2S. The c-di-GMP-dependent transcription factor VpsR activated by c-di-GMP, induces transcription of the extracellular protein secretion encoding *eps* operon and putative transcription factor encoding *tfoY* for the expression of T2SS and T6SS, respectively (Fernandez et al., 2018). In addition, T2SS also secretes the three proteins RbmA, RbmC, and Bap1, which are necessary for biofilm formation.

T3SSs are commonly detected in Gram-negative bacterial pathogens, but their mode of action and functions vary, as each species make different effector proteins. Some segments of the *V. cholerae* T3SS genomic island are analogous in gene organization and protein coding content of T3SS2 in *V. parahaemolyticus*. However, the flanking sequences are less conserved between these two species. After translocation, effector proteins of T3SS can enact a wide variety of functions to promote pathogenesis. T3SS-positive *V. cholerae* strains encode three regulators; VttR_A_, VttR_B_, and ToxR. VttR_A_ and VttR_B_ are the transmembrane transcriptional regulatory proteins encoded within the horizontally acquired T3SS genomic island, whereas ToxR is encoded on the chromosome. VttR_A_ and VttR_B_ are integral membrane proteins with N-terminal, cytoplasmic DNA binding domains and overall sequence similarity to ToxR (Miller et al., 2016a).

Most of the non-toxicgenic *V. cholerae* use T3SS as their prime virulence mechanism. The T3SS pathogenicity islands have a tripartite structure. A conserved “core” region encodes functions necessary for colonization and disease, including modulation of innate immune signaling pathways and actin dynamics, whereas regions bordering core sequences mostly encode effector proteins that perform a diverse array of activities. T3SS-mediated machinery helps the pathogen to colonize host cells and interrupt homeostasis. It was observed that T3SS induced toxicity does not progress to apoptotic or necrotic mechanisms, but displays osmotic lysis (Miller et al., 2016b). The collective effect of translocated proteins (VopS) and other effectors contributes to the colonization of T3SS-positive strains in the host epithelial cells (Chaand et al., 2015). All the three T3SS regulators are important for eukaryotic cell death as ToxR and VttR_A_ act upstream of VttR_B_ and modify the level of either *vttRA* or *vttRB* and strongly influence T3SS gene expression. Suppression of T3SS-encoded VttR regulatory proteins showed attenuated colonization *in vivo* (Alam et al., 2010).

T6SS is a contact-dependent factor, which resembles a contractile phage tail, but with an orientation that is opposite of the phage tail. It eliminates the competitors in the gut through the translocation of proteinaceous toxins. *V. cholerae* can use T6SS to damage both host cells and other members of the gut microbiota (Ho et al., 2014). T6SS genes are encoded in a major and at three auxiliary clusters. Activation of the major cluster induces transcription of more than one auxiliary clusters and the production of an assembled T6SS. The genes coding for the secreted core components (Hcp, VgrG, PAAR) are found in auxiliary clusters together with the genes that encode the effectors-immunity pairs. T6SS machinery is ATP-dependent actions involving expression of more than 10 genes. Some of these genes are regulated by the TfoY (encodes a putative transcription factor in *Vibrio* spp.) that responds to c-di-GMP levels. But, the mechanisms or external signals that induce the secretion activity is not fully known. LonA belongs to the superfamily of ATPases that repress the T6SS and influence T6SS-dependent killing phenotypes differently in *V. cholerae* strains with high and low levels of c-d-GMP (Joshi et al., 2020).

Four T6SS effectors mediate bacterial killing that includes cell membrane targeting TseL (putative lipase), VasX (pore forming colicin), peptidoglycan targeting VgrG-3 (lysozyme), and TseH (amidase) (Joshi et al., 2017). Effectors like VgrG proteins could damage substrates present in the periplasm or cytosol of target bacteria. Presence of immunity proteins prevent self intoxication by neutralizing effectors. For this, *V. cholerae* expresses an anti-toxin encoded immediately downstream of *vgrG-3* that inhibits lysis through direct interaction (Brooks et al., 2013). In addition, three protein-encoding genes *tsiV1-tsiV3* provides *V. cholerae* strains with T6SS immunity (Miyata et al., 2013).

The T6SS is controlled through the QS, catabolite repression, and nucleoside scavenging pathways that are influenced by temperature, osmolarity, c-di-GMP, mucin, and bile acid. When exposed to different environments, *V. cholerae* have distinct regulatory pathways that maintain the function of T6SS. In hosts, mucins from intestinal cells increase the ability of *V. cholerae* to kill target bacterial cells while bile acids regulate expression of T6SS genes. High osmolarity conditions induce T6SS gene expression through the osmolarity controlled regulator (OscR), while cold shock protein CspV increases expression of T6SS genes. QS and the protein TsrA (VC0070) repress T6SS. Disruption of TsrA and the LuxO induces expression and secretion of a hemolysin-coregulated protein (Hcp), which is dependent on the downstream regulator HapR. HapR binds directly to the promoter regions of the T6SS genes *hcp1* and *hcp2* to induce their expression (Zheng et al., 2010). Thus, TsrA functions as a global regulator to activate expression of hemagglutinin protease and repress CT and Tcp. In *Drosophila* model, Fast et al. (2020) have shown that *V. cholerae* lacking VasK (T6SS inner-membrane protein), or VipA (a component of T6SS outer sheath), did not prevent intestinal proliferation. T6SS genes are repressed by QS response regulator LuxO at low cell density and activated by the HapR at high cell density. Quorum regulatory small RNAs (Qrr sRNAs) in the QS cascade are required for these regulatory effects to control T6SS by repressing the expression of the large cluster through base pairing and they in turn repress HapR, the activator of the two small clusters (Shao and Bassler, 2014).

## Flagellar Regulatory System

A fully functional flagellum is required for attachment and colonization of *V. cholerae*. During the process of colonization, *V. cholerae* detaches its flagellum and penetrates the mucosal layer of the intestinal epithelium. Flagellar regulatory system controls the expression of non-flagellar genes. The loss flagellum results in the secretion of the anti-σ factor FlgM, followed by activation of another σ-factor FliA, which represses HapR, facilitating increased expression of CT and Tcp (Tsou et al., 2008). It was also shown that the major virulence genes encoding CT, Tcp, HlyA, and T6SS genes are upregulated in *V. cholerae* strains with mutated flagellar regulatory genes (*rpoN, flrA, flrC*, and *fliA*) (Syed et al., 2009). These regulatory genes are important in increased hemolysis of human erythrocytes. Derepression of virulence factor occurs concomitantly with the entry of the pathogen into the intestinal mucosal layer. Stringent response stimulated by the accumulation of guanosine pentaphosphate/tetraphosphate (p)ppGpp under stress conditions play an important role in the growth and virulence of *V. cholerae*. Administration of glucose, which is present in the oral rehydartion solution might reduce (p)ppGpp-mediated CT-production and growth of the pathogen under the influence of anaerobic glucose metabolism (Oh et al., 2016). Genome-wide screening has revealed that flagellin FlaC is involved in flagellum function and toxin production at higher (p)ppGpp levels (Kim et al., 2018). The other flagellar protein FlgT is essential for motility, attachment and colonization of *V. cholerae* (Martinez et al., 2010). In addition, the flagellar regulatory system positively regulates transcription of a diguanylate cyclase (CdgD), which regulates transcription of a hemagglutinin (*frhA*) that helps in adherence of the pathogen to chitin and epithelial cells and increases biofilm formation and intestinal colonization.

In *V. cholerae* flagellar synthesis, more than 40 gene products are known to take part. These genes belong to four classes and among these, the role of *flrD* encoding the flagellar regulatory protein D has been investigated in detail (Moisi et al., 2009). *flrD* has been transcribed independently and FlrD positively regulates class III and IV flagellar gene transcription that contributes to intestinal colonization in mice. In addition to *flr* genes, the class II operon commencing with *flhA* also encodes five chemotaxis genes (*cheY3, cheZ, cheA, cheB*, and *cheW1*). Hypervirulent *V. cholerae* strains display high levels of *flhA* expression, but decreased expression of *cheW1* needed for chemotaxis. Thus, in the life cycle of *V. cholerae*, the flagellar and chemotaxis gene cluster have different regulations. In the disease process, flagellar genes briefly repress chemotaxis genes and maintain motility to accelerate infectivity.

## Role of Histone-Like Nucleoid-Structuring (H-NS) in Repression of Virulence-Realted Genes

Inherent gene silencing controls expression several virulence factors and gut colonization of *V. cholerae*. Generally, gene silencing can occur during transcription or translation depending on the environmental factors. H^+^Cl^−^ transporter chloride channel (ClcA), which is repressed *in vivo* has a spatiotemporal expression pattern. It is induced in the stomach for acid tolerance response during stomach passage, but is silenced in the lower gastrointestinal tract by proton-motive force under alkaline pH to avoid detrimental effects and ensure better colonization (Cakar et al., 2019).

**Figure 5:**
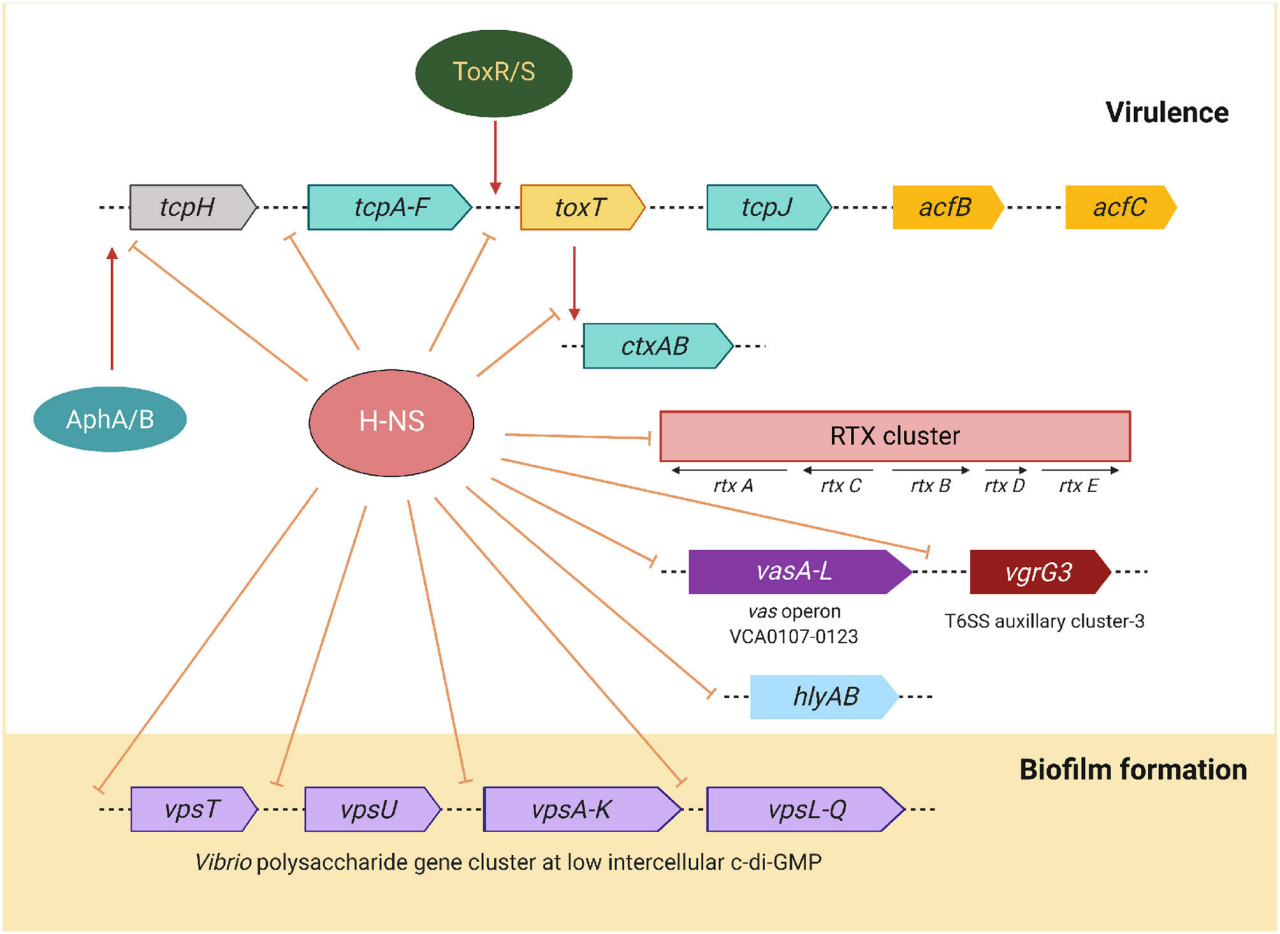
Schematic representation of *V. cholerae* H-NS mediated negative modulation virulence and biofilm expression. Apart from the *ctxAB* operon, most of the genes are silenced by H-NS is located in the *tcp, rtx* gene clusters, *vas* T6SS operon, *hlyA* hemolysin/cytolysin, and the *vps* biofilm utilization genes at low c-di-GMP. Lines with base near the intergenic regions represent H-NS repression. Red arrow indicates activation of corresponding genes.

Histone-like nucleoid-structuring (H-NS) protein belongs to a family of small nucleoid-associated proteins that are involved in the maintenance of chromosomal architecture in bacteria and play a role in silencing the expression of a variety of virulence and environmentally regulated genes (Silva et al., 2008). H-NS targets genes with a high A + T base content and inhibits their transcription by RNA polymerase. This transcription silencing activity is said to be one of the evolutionary processes that assist the pathogens to acquire new genes and integrate them into their genome. H-NS is required for adaptation of the pathogen to specific environments, including its ability to shift between different lifestyles (Ayala et al., 2017). Genes within these specific clusters are activated in response to environmental fluctuations by the action of transcription factors that negate H-NS repression. The major repression targets by H-NS are shown in Figure 5. H-NS directly represses the transcription of *hlyA* and *rtx* (Wang et al., 2015). It was also shown that H-NS silences the virulence gene expression by repressing transcription of the ToxR cascade at various levels, including *toxT, tcpA*, and *ctxA* promoters (Nye et al., 2000). Binding of the positive regulator ToxT to *ctxA* and *tcpA* promoters displaces H-NS and allows the RNA polymerase to initiate transcription (Yu and DiRita, 2002). Interestingly, the hns mutants of *V. cholerae* exhibited reduced bile- and anaerobiosis-mediated repression of *ctxA* expression and the ability to colonize in the intestine (Krishnan et al., 2004; Ghosh et al., 2006).

## Role of Bile Acids in Virulence Regulation

*V. cholerae* encounters bile acids in the early stages of infection. Seen in this light, many investigations proved the role of bile as an environmental cue to influence virulence genes (Krukonis and DiRita, 2003). *V. cholerae* uses small intestinal bile to modify the intracellular concentration of c-di-GMP which consequently impacts virulence gene expression. The ability of bile acids for stimulation and activation of *ctxAB* by ToxRS is governed by the transmembrane domain of ToxR in the inner membrane (Hung and Mekalanos, 2005). *In vitro* expression of CT by the classical vibrios in the presence of bile acids is due to the direct ToxRS activity at the *ctx* promoter and not through ToxT. Inability of bile acids to stimulate ToxRS-dependent expression of CT in El Tor biotype is related to the difference in *ctx* promoter responsiveness, which differ in the number of heptad TTTTGAT repeats in the upstream region (Hung and Mekalanos, 2005). El Tor strains carry only two to four copies of heptad repeats, whereas classical strains typically have eight repeats. The number of repeats forms binding regions for the activator, there by increasing higher CT-expression.

Depending on oxygen limitation and bile salts, cysteine residues are known to form homodimers or intramolecular disulfide bonds, which are important for the regulation or protein stability of ToxR, AphB, TcpP, and TcpH. Homodimer configuration of TcpP is important in the activation of *toxT* transcription. Bile increase TcpP homodimerization by limiting periplasmic disulfide interchange protein DsbA reoxidation that form intermolecular TcpP-TcpP disulfide bonds (Xue et al., 2016). Unsaturated fatty acids (UFAs) in bile inhibit the activity of ToxT. It was hypothesized that ToxT will be in an inactive state when it binds with UFAs in the small intestine and due to its inability to dimerize the virulence genes are repressed. Inside the mucus or epithelial cells, ToxT can dimerize and activate the expression of virulence genes due to the low concentration of bile inside the cellular milieu (Cruite et al., 2019). This complex interaction and complex virulence regulatory network require detailed investigation.

Bile acids also control the gut colonization of *V. cholerae*. Although two effective bile-regulated resistance-nodulation-division (RND)-family efflux systems, *vexAB* and *vexCD* provide high-level bile resistance, deletion of these genes was found not related to the colonization of *V. cholerae* in infant mice (Bina et al., 2006). In the gut, bile acids along with the Ca^2+^ function as host signals to activate *V. cholerae* virulence cascade through the dimerization of TcpP by inducing the formation of intermolecular disulphide bonds in its periplasmic domain (Yang et al., 2013). Ca^2+^ alone does not affect virulence, but it enhances bile salt-dependent induction of Tcp and also promotes bile salt-induced TcpP-TcpP interaction (Hay et al., 2016). Thus, Ca^2+^ and bile salts together may affect TcpP membrane movement and regulate TcpP activity.

Bile salts interact and destabilize ToxRp. Remarkably, the destabilized ToxRp augments interaction with ToxSp to promote the ToxR activity. In contrast, alkaline pH, which is one of the factors that leads to ToxR proteolysis, decreases the interaction between ToxRp and ToxSp (Midgett et al., 2017). As a consequence, *V. cholerae* alkalinizes its environment, which decreases the interaction between these two proteins and allows the ToxR proteolysis to progress. OmpT and OmpU are pore-forming outer membrane proteins of *V. cholerae*. Interaction between ToxR and ToxS has been controlled by the binding of bile acids, which increases active transcriptional complex and OmpU and OmpT expression. Bile stimulates ToxR-mediated transcription of *ompU* making *V. cholerae* more resistant to bile than the *ompT*-expressing ones (Wibbenmeyer et al., 2002). As the OmpU-OmpT system provides a channel that allows hydrophilic solutes to pass through the outer membrane, this effect indicates that bile might interfere with this traffic in OmpT-producing cells by functionally inhibiting the OmpT pore.

## Role of Quorum Sensing (QS) and Biofilm Formation

**Figure 6:**
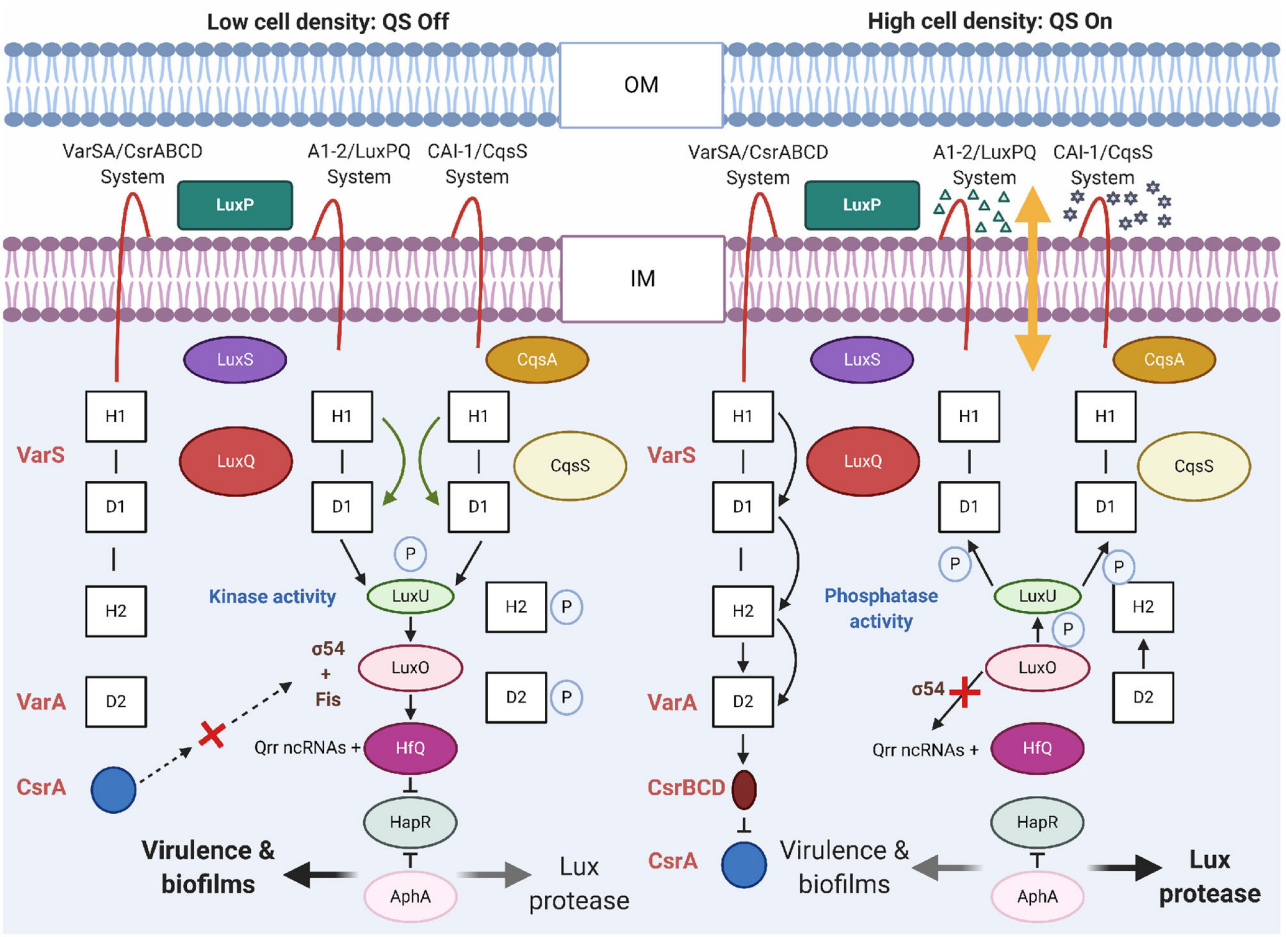
Quorum sensing circuits in *V. cholerae*. In *V. cholerae*, QS outcome depends on the cell density. At a low cell density (LCD), QS will be in a switch-off mode. With low autoinducer concentration, the membrane proteins LuxPQ and CqsS act as kinases and direct phosphate (P) through LuxO to LuxU. With σ54 of RNA polymerase, LuxO initiates transcription of genes encoding regulatory Qrr ncRNAs. The Qrr ncRNAs activate translation of AphA and with Hfq, it represses translation of HapR. The above mentioned conditions promotes expression of genes encoding virulence factors and biofilm formation. At a high cell density (HCD), the QS will be in a switch-on mode. At this phase, the autoinducers are accrued, AI-2 (Δ) and CAI-1 (⋆) bind to their respective receptors LuxPQ and CqsS. Binding of AI act as phosphatases to reverse the flow of phosphate (P) across the regulatory circuit and deactivate LuxO. As a result, the Qrr ncRNAs are not formed and hence, HapR translation is not repressed and AphA translation is not activated. HapR represses the virulence-related functions. Other receptors, VarSA/CsrABCD with unknown ligands, also transduce QS information through LuxU. DNA-binding protein Fis functions together with AI-2/LuxPQ, CAI-1/CqsS, and VarS/VarA-CsrABCD systems to increase the activity of LuxO-phosphate at LCD. At the HCD, Fis and VarS/A-CsrABCD are inactive. OM and IM, indicate outer- and inner-membrane, respectively. H and D in the boxes denote histidine and aspartate sites of phosphorylation, respectively. Dotted arrows denote hypothetical interaction.

Quorum sensing is a cell-to-cell communication leading to the synchronization of various bacterial functions, such as biofilm formation, expression of virulence, production of secondary metabolites, and competition adaptation mechanisms through secretion systems. *V. cholerae* uses QS to regulate the expression of virulence genes in response to changes in cell density. The QS circuit of *V. cholerae* consists of two autoinducer/sensor systems, cholera autoinducer-1/cholera quorum-sensing receptor (CAI-1/CqsS) and autoinducer-2 (AI-2)/LuxPQ, and the virulence associated regulator/carbon storage regulator/CsrB,C,D (VarS/VarA-CsrA/BCD) growth-phase regulatory system (Lenz and Bassler, 2007). The function of these systems depends on the *V. cholerae* cell density (Figure 6). Generally, QS is controlled by global quorum regulator, LuxO. VarS/VarA is a two-component system that acts on the phosphorelay pathway, upstream of HapR to regulate its expression.

Using QS, *V. cholerae* coordinates gene expression through the production, secretion, and detection of signaling molecules using AIs. Accumulation of AIs triggers repression of genes responsible for virulence factors and biofilm formation. These AIs function in parallel while, CAI-1 detects cell abundance, and the AI-2 allows *V. cholerae* to assess the adjacent bacterial community (Bridges and Bassler, 2019). QS is connected to virulence gene expression through AI molecules that share single signal transduction pathway to control the production of AphA, a key transcriptional activator of biofilm formation and virulence genes (Herzog et al., 2019). Expression of AphA is regulated by the HapR, which is regulated by the *V. cholerae* QS system. At a high cell density, the QS reduces intracellular AphA levels and this in turn lowers CT synthesis. At low cell densities, AphA levels increase, which activates expression of CT. The CAI-1 QS pathway is activated when less number of *V. cholerae* cells are present, whereas the AI-2 pathway is activated at a much higher cell density (HCD, Figure 6). For the latter, the response regulator LuxO does not reduce the expression of HapR but represses the expression of the virulence cascade with the activation of *tcpPH* expression by AphA and AphB (Kovacikova and Skorupski, 2002).

Histidine kinases (LuxPQ, CqsS, CqsR, and VpsS) have been identified as QS receptors that initiate virulence gene expression when the cell numbers are less (LCD, Figure 6). Any one of the receptors is sufficient for colonization of *V. cholerae* in the host small intestine (Watve et al., 2020). Detection of AIs by these receptors leads to virulence gene repression when the cell numbers are high. Recently, it was shown that the AI, QS autoinducer 3,5-dimethyl-pyrazin-2-ol (DPO) binds the receptor and transcription factor *V. cholerae* QS protein (VqmA) and this DPO-VqmA complex activates expression of a gene encoding the VqmR small RNA. Inside the host, several metabolites seem to act as an environmental cue for the expression of QS. Ethanolamine is a common metabolite in mammalian intestine, which has been used as an external signal for niche sensing and modulation of QS by *V. cholerae* using the cholera QS receptor (CqsR) (Watve et al., 2020). VqmR small RNA in turn post-transcriptionally controls many target genes by repressing translation of *ctx, rtxA, vpsT*, and *aphA*. *vpsT* encodes component required for biofilm formation and *aphA*, encodes the low cell density QS master regulator AphA (Huang et al., 2020).

Non-coding RNAs (ncRNAs) have complex regulatory roles in *V. cholerae* and constitute a dynamic system that allows communication among bacterial cells to synchronize several activities. At early stages of infection with LCD, phosphorylation of the regulator LuxO stimulates transcription of five quorum regulatory RNAs (Qrr 1–5, ncRNAs). These ncRNAs activate translation of *aphA*, the LCD master regulator, and repress translation of *hapR*, the HCD master regulator (Figure 6). At this stage, AphA activates the ToxT virulence regulon with expression of the Tcp and the CT. At the late stages of infection with HCD, the phosphorylation of LuxO is repressed and HapR is expressed (Rutherford et al., 2011; Shao and Bassler, 2012). HapR represses *aphA* transcription, production of Tcp and CT virulence factors, but activates expression of hemagglutinin/protease genes (Zhu et al., 2002).

Biofilm formation enhances the survival and persistence of *V. cholerae* in natural ecosystems and protects the pathogen during its passage through the stomach. Vibrio polysaccharide (Vps) is the major component of the biofilm matrix encoded in the *vps-I* and *vps-II* clusters (Fong et al., 2010). An intergenic region with the *rbm* gene cluster that encodes biofilm matrix proteins separates these clusters. In-frame deletions of the *vps* and *rbm* gene clusters drastically reduce the biofilm formation and intestinal colonization, indicating the functional association of these clusters.

In *V. cholerae*, c-di-GMP regulates several cellular activities. c-di-GMP and QS are important signaling systems that support *V. cholerae* in the aquatic environment and an intestinal milieu by positively regulating biofilm formation and negatively regulating virulence (Conner et al., 2017). Due to the low intracellular concentration during infection, c-di-GMP represses the expression of virulence factors. During its transmission within the host, several proteins degrade and produce c-di-GMP. This process recognizes distinct microenvironments within the gut using bile salts and bicarbonate as chemical cues and responds by modulating the intracellular concentration of c-di-GMP (Hammer and Bassler, 2009). At high c-di-GMP concentrations, c-di-GMP-dependent transcription factors VpsR and VpsT induce the expression of genes required for biofilm formation, in particular, the VPS operons and the gene cluster *rbmBCDEF* encoding biofilm matrix proteins. In addition, the c-di-GMP upregulates expression of the extracellular protein secretion (*eps*) genes encoding the T2SS via the c-di-GMP-dependent transcription factor VpsR (Sloup et al., 2017). Modulation of c-di-GMP levels, which controls biofilm formation in response to distinct sensory pathways is dependent on the *V. cholerae* biotypes. Overexpression of QS-activated HD-GYP protein (histidine [H] and/or aspartate [D] superfamily of metal dependent phosphohydrolases contain an additional GYP motif) in El Tor vibrios, decreases the intracellular concentration of c-di-GMP, which in turn reduces the exopolysaccharide production and biofilm formation. In classical vibrios, the global regulator VieA signaling pathway controls the c-di-GMP levels and biofilm formation (Hammer and Bassler, 2009).

## Other Secreted and Indigenous Regulatory Factors

Several other factors either secreted by *V. cholerae* or available in the gut milieu, aid in its survival, colonization and expression of virulence. *V. cholerae* neuraminidase releases intestinal epithelial sialic acids as a nutrition source and also modifies intestinal polysialylated gangliosides into GM1 (Alisson-Silva et al., 2018). Utilization of sialic acid as a carbon and energy source possibly helps *V. cholerae* in colonizing the mucus-rich environment of the gut. Neuraminidase may act synergistically with CT and increase the secretory response by binding and penetration of the toxin to enterocytes. Loci VC1758 to VC1809 (*nan-nag* gene cluster) present in the VPI-2 was identified to be involved in the transport and catabolism of sialic acid (Almagro-Moreno and Boyd, 2009). During the infection process, the host cells produce nitric oxide (NO), which is a toxic radical and disrupts the function of bacterial proteins. Conversely, *V. cholerae* genome encodes a NO sensor (NorR) and a NO detoxifying enzyme (HmpA). These sensor and detoxification systems play a significant role in the survival of *V. cholerae* during gut colonization (Stern et al., 2012). The host-inducible NO synthase (iNOS) is suppressed by the *V. cholerae* regulatory protein NorR, which is expressed by NO detoxification genes *hmpA* and *nnrS* under microaerobic conditions. In adult mouse colonization model, it was shown that during prolonged colonization of *V. cholerae*, both the *hmpA* and *norR* are important for the iNOS- and non-iNOS resistance (Stern et al., 2012). *V. cholerae* secretes GlcNAc binding protein A (GbpA) that acts as adherence factor and supports attachment of the pathogen to *N*-acetylglucosamine (GlcNAc)-containing carbohydrates (chitin) as well as to intestinal mucin. GbpA has been shown to enhance gut colonization and fluid accumulation in mouse models (Wong et al., 2012).

Bacteria employ specific transduction pathways in sensing and responding to a wide variety of signals. Two component systems (TCS) are signaling pathway involved in stress response. The Cpx (conjugative plasmid expression) pathway is a TCS, which exerts extracytoplasmic stress response and helps the bacteria to maintain the integrity of the cell envelope by detecting and responding to damage by changing the protein composition of the outer and inner membranes. Cpx is also associated with the regulation of envelope-localized virulence determinants (Acosta et al., 2015a). The Cpx envelope stress response is mediated by membrane-localized sensor histidine kinase CpxA and the cytoplasmic response regulator CpxR. In *V. cholerae*, activation of the Cpx pathway decreases the expression of CT and Tcp through repression of ToxT regulator and TcpP (Acosta et al., 2015b). Cpx dysfunctions Crp, which results in reduction in TcpP production.

Bicarbonate present in the upper small intestine is an important chemical stimulus that induces virulence in *V. cholerae* O1 by enhancing ToxT activity during the course of infection. The ethoxyzolamide inhibition assay has shown the conversion of CO_2_ into bicarbonate by carbonic anhydrase plays a role in virulence induction (Abuaita and Withey, 2009). Efflux systems present in *V. cholerae* remove substances detrimental to it from its cytosol. For example, RND family efflux systems help in developing antimicrobial resistance and virulence expression in *V. cholerae* (Bina et al., 2008; Taylor et al., 2012). RND-null strain expresses less CT, Tcp, and colonization in infant mouse and was also linked to the reduced transcription of *tcpP* and *toxT*. It was shown that the loss of RND efflux affected the activation state of periplasmic sensing systems, including the virulence regulator ToxR (Bina et al., 2018).

For *V. cholerae*, the human intestine is the best milieu for the maximal expression of virulence genes. TcpI is a methyl-accepting chemotaxis protein that recognize pH and influence *tcpA* transcription. The role of TcpI is important for *V. cholerae* for the penetration from the neutral pH lumen into the acidic brush border of the small intestine and pilus synthesis (Selvaraj et al., 2015). AphB activates the expression of ToxR and TcpP, which jointly control the expression of ToxT (Xu et al., 2010). Anaerobiosis enhances dimerization and the activity of transcriptional activator AphB that is required for the expression of Tcp. Under aerobic conditions, AphB is modified at the C(235) residue, which is reversible between oxygen-rich aquatic environments and oxygen-limited human hosts (Liu et al., 2011). This thiol-based switch mechanism senses intestinal signals and activates virulence. Other chromosomally encoded putative virulence related genes/proteins are provided in Supplementary Table 1.

## Regulation of Invasive Mechanisms

*V. cholerae* O1/O139 colonizes in the small intestine, but does not invade the intestinal tissue (i.e., noninvasive). *V. cholerae* non-O1, non-O139 serogroups occasionally can cause extraintestinal disease, mostly through their invasive mechanisms. In *V. cholerae*, the invasive mechanisms are complex and multifactorial. During the different stages of infection, regulation of an extracellular pore-forming hemolysin encoding gene (*hlyA*) by HapR, Fur, and HlyU was considered advantageous to the invasion of *V. cholerae*. However, the pathogenesis of invasive infections caused by these vibrios have not been fully investigated. Patients with predisposing factors such as cirrhotic condition and thrombocytopenia are susceptible to *V. cholerae* non-O1, non-O139 bloodstream invasion, bacteremic skin, and soft tissue infections including necrotizing fasciitis (Lee et al., 2007; Maraki et al., 2016). Remarkably, majority of these vibrios have *tcpA* and that could suggest its role in the pathogenesis. In some of the clinical cases, it was shown that the *V. cholerae* non-O1 and non-O139 causes invasive infection using the Zot by increasing the permeability of epithelial barrier and destabilizing the TJs junctions (Kharlanova et al., 2004). *Vibrio parahaemolyticus* has a second T3SS2 in its chromosome-II that mediates invasion into non-phagocytic cells using an effector, VopC that has a deamidase/transglutaminase activity (Park et al., 2004). Some of the pathogenic *V. cholerae* non-O1, non-O139 strains shown to contain a T3SS2-like gene cluster with the VopC and was found to be responsible for the invasion in HeLa cells (Zhang et al., 2012).

## Host Response

*V. cholerae* evokes long lasting immunity, responding to several antigens, in the host. During the infection process, there is a strong interaction between the host and the pathogen. At first, a number of non-specific defense mechanisms present in the host come into play, which is followed by a strong immune response mounted by the host. To initiate infection, *V. cholerae* must elude the host intestinal innate immune system; breach the mucus layer of the small intestine, adhere, and proliferate on the surface of microvilli and produce toxin(s) through the action of virulence encoded genes. In the *in vivo* model, CT-induced intestinal barrier disruption and TLR-4-NF-κB-mediated COX-2 expression has been illustrated in the pathogenesis of *V. cholerae* O1. It has been found small intestinal epithelial cells expresses the immunomodulatory microRNAs at the acute stage of infection. *V. cholerae*, like other Gram-negative bacteria discharges spherical membrane-enclosed virulence molecules called OMVs that help it to translocate its cytolysin by inducing immunomodulatory micro-RNAs (miR-146a) for colonization, reducing the epithelial innate immune defense system and preventing inflammation in the mucosa (Bitar et al., 2019).

### Response to Toxins and Somatic Antigens

CT is the prime virulence factor of *V. cholerae*, but the antitoxin responses to CT do not induce long-term protective immunity against cholera. Household cholera contact studies indicated that CtxB-IgG antibodies and CtxB-specific memory B cells do not play a role in providing protective immunity, whereas elevated levels of CtxB-IgA have shown to be associated with protection (Harris et al., 2008). However, serum IgA wanes rapidly after natural infection (Harris et al., 2009). It was also shown that Ctx-B activates immune cells by inducing interleukin-1β production from the peritoneal macrophages via the pyrin inflammasome as well as the nucleotide-binding domain (NOD)-like receptor protein 3 (NLRP3) inflammasome (Orimo et al., 2019). In several cholera vaccine trials, immunomodulatory role of Ctx-B was shown to give higher protective efficacy.

Somatic antigen (O)-specific polysaccharide antigen (OSP) immunity has been maintained for long in the memory B-cell compartment at the mucosal surface. Hence, host OSP antibody responses are more important in protection against cholera than the CT antibodies. The main responses of OSP antibodies include, inhibition of motility of OSP-targeted IgA antibodies by interfering with flagellar function and pathogen trapping and elimination preceding the colonization of the small intestine (Levinson et al., 2016; Harris, 2018). Serum vibriocidal responses have long been considered as a best surrogate marker for immuno-protection upon vaccination (Chen et al., 2016; Ritter et al., 2019). Though strong vibriocidal responses are evident in children below 5 years of age, vaccine efficacy was found to be low with shorter duration of protection (Ritter et al., 2019).

Generally, *V. cholerae* infection may not trigger clinically overt inflammation, but disruption of intestinal homeostasis for extended duration may be associated with long-lasting cholera immunity (Bourque et al., 2018). Some of the innate signaling pathways upregulated in response to *V. cholerae* infection are not similar to the innate immune response to other bacterial infections. For example, in cholera, the nucleotide-binding domain leucine-rich repeat pyrin domain-3 (NLRP3) inflammasome, and type I interferon signaling pathways are activated to a level similar to viral infections (Bourque et al., 2018). Innate immunity functions as a primary defense, but in severe cholera this protection may be ineffective. The innate immune system recognizes *V. cholerae* and generates signals to direct T- and B-lymphocytes. This immune modulation is accomplished through the increased production of cytokines, including interleukin-1β (IL-1β), IL-6, and IL-17. An innate signaling pathway activated during infection process is to induce the expression of proteins that produce ROS. Dual oxidase-2 and inducible nitric oxide synthase are some of the upregulated proteins identified in duodenal tissue during *V. cholerae* infection (Bourque et al., 2018). Nontoxigenic El Tor vibrios infection is distinguished by the upregulation of IL-6, IL-10, and macrophage inflammatory protein-2α in the intestine, indicating an acute innate immune response. From several reports, it could be seen that the innate immune system directs the progression of subsequent adaptive immunity (Weil et al., 2019).

Phosphate, a component of nucleotides plays an important role in biological systems serves as an energy repository within cells (ATP) or in linking nucleotides together to form nucleic acids. It is also an important component of membrane phospholipids that is incorporated into proteins during post-translational modifications, mainly as a regulatory tool, which plays a role in signal transduction of TCS. At the high phosphate conditions of the gut, the AphA/AphB regulatory cascade promotes Tcp and CT-production. At low phosphate conditions, the transcriptional-response regulator (PhoB) controls the *V. cholerae* virulence regulation cascade by binding and repressing the *tcpPH* promoter (Chekabab et al., 2014).

### Inflammatory Response

Unlike O1/O139 serogroups, the nontoxigenic *V. cholerae* generally induces gastritis. *V. cholerae* colonizes in the small intestine and along with other factors causes acute inflammation. The mRNA expression of pro-inflammatory and anti-inflammatory cytokines revealed coordinated activity of up-regulation of IL-1α, IL-6, granulocyte-macrophage colony-stimulating factor (GM-CSF), monocyte chemoattractant protein-1 (MCP-1) and down-regulation of TGF-β in *V. cholerae* infected Int407 cells. It is evident that the whole action is modulated by NF-κ-B and in part by the adherence or motility of *V. cholerae* (Bandyopadhaya et al., 2007). *V. cholerae* mediates induction of host cell nuclear responses through signal transduction pathway and activation of proinflammatory cytokines in cultured intestinal epithelial cells. This cellular infection result in the activation of extracellular signal-regulated kinases and p38 of the mitogen activated protein kinase (MAPK) family. The intracellular infection activates protein kinase A (PKA) and protein tyrosine kinase (PTK) in the upstream of MAPK and NF-κB pathway (Bandyopadhaya et al., 2009a).

The outer membrane protein OmpU, which is one of the major porins in *V. cholerae*, has been shown to play an important role in intestinal inflammation by inducing IL-8 expression at the mRNA and protein levels in human intestinal epithelial cell line (Yang et al., 2018). Only the apical exposure to OmpU induce IL-8 secretion in polarized HT-29 cells. Mitochondria play a crucial role in the OmpU-mediated cell death. OmpU translocate to the mitochondria and directly initiates membrane permeability modifications and apoptosis-inducing factor release, which probably opens the mitochondrial permeability transition pore (Gupta et al., 2015).

*V. cholerae* is affected by host-derived hormones and neurotransmitters, the adrenaline and non-adrenaline signaling molecules. These molecules modulate the growth and virulence of the pathogen. Adrenaline transformed by *V. cholerae* to adrenochrome during the process of its respiration not only stimulates the growth of the pathogen, but also elicits specific responses in immune cells (Toulouse et al., 2019). In the host, adrenochrome inhibited lipopolysaccharide activates formation of TNF-α by THP-1 monocytes. The adrenochrome formed from adrenaline thus functions as an effector molecule during pathogen-host interaction. In addition, stress-associated hormones epinephrine and norepinephrine of the human host also act as signal molecules to support *V. cholerae* growth by the sequestration of the host's iron and production of virulence factors (Halang et al., 2015).

As mentioned before, GbpA is a secretory protein of *V. cholerae* that accelerates its adherence to human intestine. Binding of *V. cholerae* to intestinal mucin by GbpA results in increased mucus production, which draws more bacteria for better colonization (Rothenbacher and Zhu, 2014). GbpA interaction causes accumulation of reactive oxygen species that leads to mitochondrial dysfunction, relocation of NF-κB into nucleus, and necrotic response of the host cells (Mandal and Chatterjee, 2016). Protein kinase-B, also known as Akt, is a serine/threonine-specific protein kinase that plays a key role in the regulation of cellular survival. In *V. cholerae* infected Int cells, inhibition of Akt significantly decreases IL-1α, IL-6, and TNF-α by the NF-κ-B activation (Bandyopadhaya et al., 2009b).

The disease processes and the host responses to infection caused by toxigenic and non-toxigenic *V. cholerae* are inherently different. Infection caused by the non-toxigenic *V. cholerae* is characterized by involvement of extraintestinal organs, and a systemic inflammatory response mostly associated with immunosuppression, which induces more inflammatory gastrointestinal symptoms (Queen and Satchell, 2013). The role of neutrophils is to protect the host against pathogens and limit the infection to the intestine and control its spread to extraintestinal organs. They also control the levels of IL-1β and tumor necrosis factor-α. Invasive infections caused by non-toxigenic *V. cholerae* is characterized by upregulation of interleukin-6 (IL-6), IL-10, and macrophage inflammatory protein-2α in the intestine (Queen and Satchell, 2012). Strong evidence for this comes from the observation that reducing neutrophils from mice with anti-Ly6G-IA8 (a member of the Ly-6 superfamily of glycosylphosphatidylinositol-anchored cell surface proteins with roles in cell signaling and cell adhesion) monoclonal antibody led to decreased survival of mice infected by non-toxigenic *V. cholerae*. However, it seems that neutrophils are not protective during infection with a CT-expressing strains (Queen and Satchell, 2013).

Stimulation of the inflammasome in human THP-1 monocytes and in primary human peripheral blood mononuclear cells (PBMCs) is mediated by both nucleotide-binding oligomerization domain-like receptor family pyrin domain-containing 3 (NLRP3)-dependent and -independent pathways. However, the degree of stimulation depends on *V. cholerae* biotypes. El Tor biotype induces release of interleukin-1β (IL-1β) dependent on NLRP3 and apoptosis-associated speck-like protein-containing a caspase recruitment domain (ASC), due to the secreted pore-forming toxin hemolysin. Classical biotype strains do not produce either hemolysin or the MARTX toxin. Induction of low-level IL-1β release is due to CT and dependent on ASC but independent of NLRP3 and pyroptosis (Queen et al., 2015).

## Influence of Microbiome on *V. cholerae* Pathogenesis

Symptoms of cholera is essentially due to CT, which *V. cholerae* elaborates after it colonizes the human gut, a place where millions of other microbes reside and interact. Recently, the importance of gut microbiome has been recognized for their role in controlling the enteric infections as they provide colonization resistance, nutrient competition, secretion of antimicrobial compounds, gut barrier integrity, mucosal adjuvant activity, etc. The disease cholera eliminates the normal gut microbes in two ways. First, the efflux of secreted water and salts into the intestinal lumen removes the protective mucus along with the gut microbiota. Second, the reduction of the microbiota may be exacerbated large amounts of oral rehydration solution used in the treatment of cholera and T6SS by the pathogen.

In gnotobiotic mice model, improved CT-IgA response was observed after colonizing with diet-specific microbiome (Di Luccia et al., 2020). In several studies, it was shown that the commensal microbes act as a barrier against enteric pathogens. In grem-free adult mice, colonization of *Bacteroides vulgatus* was shown to reduce *V. cholerae* colonization after conditional challenge (You et al., 2019). On the other hand, some microbes make gut as a conducive milieu for the proliferation of enteric pathogens through their metabolic process. In the adult mice model, it was shown that a decrease of ROS in the gut by the non-lactose fermenting *E. coli* support the growth of *V. cholerae* (Yoon et al., 2016). The host derived metabolites also play a role in supporting the such pathogens in the gut, but the studies focused on this line are very less.

Gut microbial composition determines susceptibility to *V. cholerae* infection and can be used as a epidemiologic tool to predict risk factors (Midani et al., 2018). With the development of large scale microbial sequencing, microbiome studies are increasingly available. It has been shown that in case of cholera, rapid loss of normal flora occurs due to massive loss of body fluids through the intestine and/or due to the influence of antibiotic treatment. Restoration of normal gut microbiota requires bacterial species succession after cholera infection, which has been demonstrated with different stages. In all these stages, relative abundance of oxygen tension and nutrient availability play a crucial role (David et al., 2015). Knowledge of the role of gut microbes that impact host-pathogen interactions in *V. cholerae* infection increasing. Investigations of the specific bacterial groups associated with susceptibility may provide understanding of relationships between *V. cholerae* infection and the gut microbiome. Considering several benefits provided by gut microbes and the dynamic nature of the gut environment, use of microbiome in the development of therapeutic strategies.

## Conclusion

As this review has attempted to highlight virulence regulation and host responses during the infection process of *V. cholerae*. The risk of infections associated with not just *V. cholerae*, but also other *Vibrio* species is increasing, as a consequence of unhealthy environment, food contamination and global warming. *V. cholerae* has several virulence factors, and for some of them, the mechanisms associated with the infections are not completely understood. Horizontal gene transfer plays a significant role in the transmission of genes encoding the toxins. This mechanism constantly changes the phenotypic and genetic characteristics of *V. cholerae*. As far as the virulence is concerned, the role of RNA-mediated regulation factors and specific ncRNA population that regulates different target molecules are not known at the single-bacterial cell level. Mechanisms by which the gut microbes interact with *V. cholerae* remain unknown. Based on the voluminous data that we have in hand, trans-disciplinary approaches using pan-omic technologies have to be developed to recognize patho-adaptation, environmental signals and host responses that participate in the genetic regulatory networks. The information on these factors is crucial for developing novel therapeutics and prevention strategies against *V. cholerae* mediated infections.

## Author Contributions

TR, RN, AM, and AG conceived and structured the review. TR and RN wrote the manuscript. AKM, SD, KO, S-IM, and GN critically analyzed the presentation. All authors discussed the results, reviewed, and commented on the manuscript.

## Conflict of Interest

The authors declare that the research was conducted in the absence of any commercial or financial relationships that could be construed as a potential conflict of interest.
