# Supplementary material for: Virulence Regulation and Innate Host Response in the Pathogenicity of Vibrio cholerae
Source: Front Cell Infect Microbiol. 2020 Sep 30;10:572096. doi: 10.3389/fcimb.2020.572096 (PMC7554612; doi:10.3389/fcimb.2020.572096)
Supplement: Supplementary file 1 [file Table_1.DOCX]

**Supplement Table 1.**

| **Gene/Protein** | **Basic function** | **Activity** | **Remarks** | **Reference** |
| --- | --- | --- | --- | --- |
| *galU* | Galactose catabolism | Forms outer membrane with  barrier function | Supports the bacteria to resist short-chain organic acids, cationic antimicrobial  peptides, the complement system, bile salts and hydrophobic  agents | Nesper et al., 2001 |
| *varA* | Virulence associated regulator | Controls transcription of *tcpA* and production of CT | In classical vibrios, this function is more distinct | Wong et al., 1998 |
| *acfA* | Accessory  colonization factor | Colonization |  | Hughes et al., 1995 |
| *acfB* | Accessory colonization factor | Colonization | Environmental  sensor/signal-transducing protein involved in the gut colonization. | Everiss et al, 1994 |
| *malQ* and *malF* | Maltose regulon | Virulence factor in infant mice | Mutants have shown reduced virulence | Lång et al., 1994 |
| *ppsA* and *pckA* | Phosphoenolpyruvate synthase and phosphoenolpyruvate carboxykinase | Gluconeogenesis | Colonization | Wang et al., 2018 |
| *fadR* | Fatty acid degradation Regulon | Influences the transcription and posttranslational regulation of  ToxT by distinct mechanisms. |  | Kovacikova et al., 2017 |
| *chiS* | Sensor histidine kinase | Colonization,  expression of *ctxA*, *toxT* and *tcpA* |  | Chourashi et al., 2016 |
| *csrA* | Carbon storage regulator | Linking environmental sensing to the ToxR, CsrA acts as a switch to control pathogenesis | CsrA is a positive regulator of ToxR | Mey et al., 2015 |
| *ptsI* and  *ptsH* | Phosphoenolpyruvate phosphotransferase system | Reduced  expression of TcpA, CT  and gut colonization in the infant mice |  | Wang et al., 2015 |
| *vxrB* | *V. cholerae* response regulator | Influence colonization  through the regulation of T6SS |  | Cheng et al., 2015 |
| CpxR | Cytoplasmic response regulator | Cpx response affects cyclic AMP receptor protein  function, which activates TcpP, ToxT, CT, and TCP |  | Acosta et al., 2015 |
| VttR(A) and VttR(B) | Transmembrane transcriptional regulators | Colonization in vivo and help bile-dependent T3SS gene  expression in vitro. |  | Chaand and Dziejman, 2013 |
| *tehAVc* | LysR-family regulator VC2324 | in vivo colonization |  | Pei et al., 2013 |
| vieA | c-di-GMP phosphodiesterase | Upregulation of t*oxT* expression with reference to host cell-adherence |  | Dey et al., 2013 |
| *tsaB* | T6SS antitoxin-B | Inhibit valine-glycine repeat G-proteins  (VgrG-3) of T6SS in a toxin-antitoxin manner |  | Brooks et al., 2013 |
| MetR and GlyA1 | Metabolic transcriptional activator and serine hydroxymethyltransferase | in vivo colonization |  | Bogard et al., 2012. |
| *hmg* | High-mobility group gene for pigment-production | Higher expression of CT, Tcp and gut colonization |  | Valeru et al., 2009 |
| *ihf* | Integration host factor | Controls the expression of CT and TcpA |  | Stonehouse et al., 2008 |
| *crp* | cAMP-receptor  protein | Regulated  CAI-1 and the expression of multiple HapR-regulated  genes |  | Liang et al., 2007 |
| *relA* | *V. cholerae* relaxed  guanosine pentaphosphate synthase-I | Controls the levels of (p)ppGpp in the cell | Mutants have shown reduced virulence with reference to production of CT and TCP | Haralalka et al., 2003 |
| *vieS* | Sensor kinase of the three component *vieSAB* signal transduction system | Essential for CT regulation |  | Tischler et al., 2002 |
| *gshB* | Glutathione synthetase | Colonization |  | Merrell et al., 2002 |
| *hepA* | Unknown/RNAP associated | Colonization |  | Merrell et al., 2002 |
| *recO* | DNA repair | Colonization |  | Merrell et al., 2002 |
| *rpoN* | σ54 | Colonization | Glutamine synthetase expression | Klose and Mekalanos, 1998 |

**Reference**

Acosta, N., Pukatzki, S., and Raivio, T. L. (2015). The Cpx system regulates virulence gene expression in *Vibrio cholerae*. *Infect. Immun.* 83;2396-2408. doi: 10.1128/IAI.03056-14.

Bogard, R. W., Davies, B. W., and Mekalanos, J. J. (2012). MetR-regulated *Vibrio cholerae* metabolism is required for virulence. *mBio.* 3; pii: e00236-12. doi: 10.1128/mBio.00236-12

Brooks, T. M., Unterweger, D., Bachmann, V., Kostiuk, B., and Pukatzki S. (2013). Lytic activity of the *Vibrio cholerae* type VI secretion toxin VgrG-3 is inhibited by the antitoxin TsaB. *J. Biol. Chem.* 288;7618-7625. doi: 10.1074/jbc.M112.436725

Chaand, M., and Dziejman, M. (2013). *Vibrio cholerae* VttR(A) and VttR(B) regulatory influences extend beyond the type 3 secretion system genomic island. *J. Bacteriol.* 195;2424-2436. doi: 10.1128/JB.02151-12

Cheng, A. T., Ottemann, K. M., and Yildiz, F. H. (2015). *Vibrio cholerae* response regulator VxrB controls colonization and regulates the type VI secretion system. *PLoS Pathog.* 11;e1004933. doi: 10.1371/journal.ppat.1004933

Chourashi, R., Mondal, M., Sinha, R., Debnath, A, Das. S., Koley, H., et al. (2016). Role of a sensor histidine kinase ChiS of *Vibrio cholerae* in pathogenesis. *Int. J. Med. Microbiol.* 306;657-665. doi: 10.1016/j.ijmm.2016.09.003

Dey, A. K., Bhagat, A., and Chowdhury, R. (2013). Host cell contact induces expression of virulence factors and VieA, a cyclic di-GMP phosphodiesterase, in *Vibrio cholerae*. *J. Bacteriol.* 195;2004-2010.  doi: 10.1128/JB.02127-12

Everiss, K. D., Hughes, K. J., Kovach, M. E., and Peterson, K. M. (1994). The *Vibrio cholerae acfB* colonization determinant encodes an inner membrane protein that is related to a family of signal-transducing proteins. *Infect. Immun.* 62;3289-3298.

Haralalka, S., Nandi, S., and Bhadra, R. K. (2003). Mutation in the *relA* gene of *Vibrio cholerae* affects in vitro and in vivo expression of virulence factors. *J. Bacteriol*. 185;4672-4682. doi: 10.1128/jb.185.16.4672-4682.2003

Hughes, K. J., Everiss, K.D., Kovach, M. E., and Peterson, K. M. (1995). Isolation and characterization of the *Vibrio cholerae acfA* gene, required for efficient intestinal colonization. *Gene*. 156;59-61. doi: 10.1016/0378-1119(95)00054-a.

Klose, K. E., and Mekalanos, J. J. (1998). Distinct roles of an alternative sigma factor during both free-swimming and colonizing phases of the *Vibrio cholerae* pathogenic cycle. *Mol. Microbiol.* 28;501-520. doi: 10.1046/j.1365-2958.1998.00809.x

Kovacikova, G., Lin, W., Taylor, R. K., and Skorupski, K. (2017). The fatty acid regulator FadR influences the expression of the virulence cascade in the El Tor biotype of *Vibrio cholerae* by modulating the levels of ToxT via two different mechanisms. *J. Bacteriol.* 99;pii: e00762-16. doi: 10.1128/JB.00762-16.

Lång, H., Jonson, G., Holmgren, J., and Palva, E. T. (1994). The maltose regulon of *Vibrio cholerae* affects production and secretion of virulence factors. *Infect. Immun.* 62; 4781-4788.

Liang, W., Pascual-Montano, A., Silva, A. J., and Benitez, J. A. (2007). The cyclic AMP receptor protein modulates quorum sensing, motility and multiple genes that affect intestinal colonization in *Vibrio cholerae*. *Microbiol.* 153;2964-2975. doi: 10.1099/mic.0.2007/006668-0

Merrell, D. S., Hava, D. L., and Camilli, A. (2002). Identification of novel factors involved in colonization and acid tolerance of *Vibrio cholerae*. *Mol. Microbiol.* 43;1471-1491. doi: 10.1046/j.1365-2958.2002.02857.x

Mey, A.R., Butz, H. A., and Payne, S. M. (2015). *Vibrio cholerae* CsrA regulates ToxR levels in response to amino acids and Is essential for virulence. mBio. 6;e01064. doi: 10.1128/mBio.01064-15

Nesper, J., Lauriano, C. M., Klose, K. E., Kapfhammer, D., Kraiss A., and Reidl, J. (2001). Characterization of *Vibrio cholerae* O1 El tor *galU* and *galE* mutants: influence on lipopolysaccharide structure, colonization, and biofilm formation. *Infect. Immun.* 69;435-445. doi: 10.1128/IAI.69.1.435-445.2001.

Pei B, Wang Y, Katzianer DS, Wang H, Wu H, Zhong Z., et al. (2013). Role of a TehA homolog in *Vibrio cholerae* C6706 antibiotic resistance and intestinal colonization. *Can. J. Microbiol.* 59;136-139. doi: 10.1139/cjm-2012-0673

Stonehouse, E., Kovacikova, G., Taylor, R. K., and Skorupski K. (2008). Integration host factor positively regulates virulence gene expression in *Vibrio cholerae*. *J. Bacteriol*. 190;4736-4748. doi: 10.1128/JB.00089-08

Tischler, A. D., Lee, S. H., and Camilli, A. (2002). The *Vibrio cholerae* *vieSAB* locus encodes a pathway contributing to cholera toxin production. *J. Bacteriol.* 184;4104-4113. doi: 10.1128/jb.184.15.4104-4113.2002

Valeru, S., Rompikuntal, P. K., Ishikawa, T., Vaitkevicius, K., Sjöling, A., Dolganov, N., et al. (2009). Role of melanin pigment in expression of *Vibrio cholerae* virulence factors. *Infect Immun.* 77;935-942. doi: 10.1128/IAI.00929-08

Wang, Q., Millet, Y. A., Chao, M. C., Sasabe, J., Davis, B. M., and Waldor, M. K. (2015). A genome-wide screen reveals that the *Vibrio cholerae* phosphoenolpyruvate phosphotransferase system modulates virulence gene expression. *Infect. Immun.* 83;3381-3395. doi: 10.1128/IAI.00411-15

Wang, J., Xing, X., Yang, X., Jung, I. J., Hao, G., Chen Y., et al. (2018). Gluconeogenic growth of *Vibrio cholerae* is important for competing with host gut microbiota. *J. Med. Microbiol.* 67;1628-1637. doi: 10.1099/jmm.0.000828.

Wong, S. M., Carroll, P. A., Rahme, L. G., Ausubel, F. M., and Calderwood SB. (1998). Modulation of expression of the ToxR regulon in *Vibrio cholerae* by a member of the two-component family of response regulators. *Infect. Immun.* 66;5854-5861.
